# A Scalable and Cost‐Effective In‐Line Barcoding Strategy for Standardized 16S rRNA Gene Amplicon Sequencing: Performance Evaluation and Bias Assessment

**DOI:** 10.1111/1755-0998.70138

**Published:** 2026-05-11

**Authors:** Lisa Jourdain, Pierre Rossi, Aline Charpagne, Emmanuelle Chevalier, Viviane Praz, Julien Marquis, Johann Weber, Wenyu Gu

**Affiliations:** ^1^ MICROBE Laboratory, Institute of Environmental Engineering, School of Architecture, Civil and Environmental Engineering Swiss Federal Institute of Technology in Lausanne (EPFL) Lausanne Switzerland; ^2^ Central Environmental Laboratory, School of Architecture, Civil and Environmental Engineering Swiss Federal Institute of Technology in Lausanne (EPFL) Lausanne Switzerland; ^3^ Lausanne Genomic Technologies Facility (GTF), Faculty of Biology and Medicine University of Lausanne Lausanne Switzerland

**Keywords:** 16S rRNA sequencing, high‐throughput amplicon sequencing, in‐line barcoding, microbiome

## Abstract

In‐line barcoding offers a streamlined and scalable alternative to two‐step PCR library preparation for 16S rRNA gene amplicon sequencing, enabling cost‐effective, high‐throughput profiling of microbial communities. Here, we tested 136 and 156 in‐line barcoded primer pairs for bacterial and archaeal communities for their performance across environmental samples and a mock standard community. The primers were designed by combining widely used universal 16S rRNA gene primers with existing barcode sets from Illumina kits. The designed primer pairs produced efficient and consistent amplification with minimal dropout and no systematic taxonomic bias. Through clustering and performance‐based filtering, we selected final sets of 96 pairs for both bacterial and archaeal communities that work efficiently and well together for direct further use. This in‐line tagging strategy is easy to adopt with fewer processing steps and PCR‐associated artefacts, allows straightforward sample tracking, and supports reliable large‐scale microbiome studies. We also present a framework for evaluating barcode‐ or primer‐induced biases. More broadly, the proposed in‐line barcoding strategy can be adapted to any amplicon‐sequencing application, as well as targeted sequencing, highlighting its relevance beyond 16S rRNA gene surveys. All validation datasets, open‐source processing scripts, and barcode design resources are provided to promote reproducibility and community‐wide adoption.

## 1 Introduction

The 1980s saw the emergence of amplicon‐based sequencing of taxonomic marker genes as a fundamental approach in microbial ecology (Giovannoni et al. 1990; Woese and Fox 1977). It provides nowadays a fast and affordable method for studying microbial communities in a wide range of environmental and host‐associated biosystems. As a culture‐independent technique, 16S ribosomal RNA (rRNA) gene amplicon sequencing circumvents biases inherent to traditional microbiology, enabling comprehensive assessments of microbial diversity (Hugenholtz et al. 1998; Pace 1997). The 16S rRNA gene has been extensively used as a phylogenetic marker since the pioneering work by Woese and Fox (Woese and Fox 1977). Its unique structure, comprising both conserved and hypervariable regions, enables broad taxonomic coverage across Bacteria and Archaea (Weinroth et al. 2022). 16S rRNA gene sequencing has become a routine method for profiling microbial community composition and diversity, providing insights into ecological interactions (Lozupone et al. 2012; Thompson et al. 2017). Over the past decades, advances in high‐throughput sequencing technologies and the development of accessible bioinformatics pipelines have democratized data generation and analysis, enabling researchers to drive the rapid expansion of ecological and biodiversity research. Despite the increasing adoption of shotgun metagenomics, 16S rRNA gene amplicon sequencing remains widely employed for taxonomic profiling due to its affordability, high‐throughput capacity, and robust performance in resolving community structure across broad taxonomic levels (Caporaso et al. 2010; Knight et al. 2018; Thompson et al. 2017).

The outputs of 16S rRNA sequencing analyses are sensitive to a variety of methodological variables, each of which can introduce systematic biases that distort taxonomic resolution and relative abundance estimates. One of the main sources of bias is the presence of mismatches between primers and templates, as even a difference of a single nucleotide can significantly reduce amplification efficiency and lead to under‐representation of specific taxa (Green et al. 2015; Kebschull and Zador 2015; Piñol et al. 2015; Sipos et al. 2007). Additional amplification biases can arise from factors like GC content, homopolymer runs, and secondary structure of the primers, all of which affect annealing and extension dynamics (Aird et al. 2011; Benjamini and Speed 2012; Best et al. 2015; Laursen et al. 2017; Moinard et al. 2023; Pinto and Raskin 2012; Suzuki and Giovannoni 1996). The choice of hypervariable region, primer set, and amplicon length can likewise influence both the accuracy of taxonomic assignments and comparability across studies (Abellan‐Schneyder et al. 2021; Darwish et al. 2021; Geisen et al. 2019; Park et al. 2021; Regueira‐Iglesias et al. 2023). Moreover, sources of variability extend beyond primer design to include DNA extraction kits and protocols (Baer et al. 2024; Brooks et al. 2015; Elie et al. 2023), the choice of the DNA polymerase (Acinas et al. 2005; Oyola et al. 2012), template concentration (Chandler et al. 1997), and PCR cycling conditions (Drengenes et al. 2021; Kennedy et al. 2014). Finally, the selection of pipelines, parameters, and filtering thresholds during bioinformatic processing can further shape the resulting community profiles (Anslan et al. 2018; Bars‐Cortina et al. 2023; Chiarello et al. 2022; Hakimzadeh et al. 2024). Collectively, these factors highlight the critical need for methodological transparency and standardization in 16S rRNA gene amplicon sequencing studies of microbial communities.

To address the need for high‐throughput yet reliable microbiome profiling, researchers have turned to sample multiplexing strategies, in which nucleotide barcodes or indexes are appended to each sample to allow pooled sequencing and subsequent demultiplexing (Bystrykh 2012). Early methods used single indexing but were soon replaced by dual‐indexing schemes, which enabled hundreds of samples to be sequenced simultaneously. Dual‐indexing strategy evolved into three primary approaches for barcoding and library construction (Bohmann et al. 2022; Taberlet et al. 2012).

In the *one‐step PCR* method, fusion primers combine the target‐specific region, sample‐specific tag, and/or sequencing adapter in a single molecule, allowing amplification and library construction in a unique reaction (Bohmann et al. 2022). While this strategy minimizes contamination risk and index misassignment, it requires the synthesis of long primers, often exceeding 80 nucleotides (Elbrecht and Leese 2015). This can reduce PCR efficiency and elevate the risk of forming chimeric artefacts, as fusion primers—with long, similar sequences at their 5′ ends—are co‐amplified within the same reaction (Bohmann et al. 2022; Smyth et al. 2010). Despite these challenges, it has been successfully applied in various metabarcoding studies (Elbrecht and Leese 2015; Hardy et al. 2017; Kozich et al. 2013; Sickel et al. 2015). The *two‐step PCR* method on the other hand, separates target amplification from index incorporation. A first PCR uses universal primers with moderate‐length overhangs, followed by a second PCR to append indexed adapters. This is the foundation of the Illumina protocol, which offers greater flexibility for primer reuse and modular design (Baym et al. 2015; Bohmann et al. 2022; Glenn et al. 2019). However, this approach requires additional handling and amplification steps, which can increase chimera formation, contamination risk, and index misassignment—though the latter can be mitigated using unique dual indexes (UDIs) (Bronner and Quail 2019; Sinha et al. 2015; Zizka et al. 2019). Finally, the *in‐line barcoding (or tagged PCR)* strategy appends short (typically 5–10 bp) sample‐specific tags to primers during the initial amplification. These fusion primers, referred to as in‐line barcodes, or tagged or barcoded primers, enable sample multiplexing. Sequencing adapters are then ligated onto pooled PCR products, with or without a subsequent PCR. This method is cost‐effective and scalable for high‐throughput studies but carries a risk of tag‐jumping when blunt‐ending with T4 DFNA polymerase and post‐ligation PCR are used in the library preparation workflow (Bohmann et al. 2022; Carøe and Bohmann 2020). While post‐ligation PCR is often included to enrich libraries and facilitate quality control and quantification for sequencing instrument loading, this step is not strictly necessary when pools of tagged amplicons provide sufficient DNA and indexed adapters are used directly (Carøe and Bohmann 2020; Schnell et al. 2015).

While the two‐step PCR protocol remains widely used in commercial and academic settings, it can introduce technical artefacts as previously described (Karst et al. 2021; Smith and Peay 2014). These issues become more apparent when working with low‐biomass or degraded samples, where the quantity and quality of the DNA are limited. Additional amplification cycles may disproportionately amplify contaminants or errors (Eisenhofer et al. 2019; Minich et al. 2019; Weyrich et al. 2019). Consequently, there is growing interest in streamlined workflows that reduce sample handling and amplification steps, thereby minimizing the risk of contamination and preserving data integrity (Callahan et al. 2016; Karst et al. 2021; Villette et al. 2021). The in‐line PCR strategy addresses these needs by enabling efficient multiplexing through a simple and scalable design. By combining 8 forward and 12 reverse barcoded primers, it is possible to label 96 samples using only 20 different primers uniquely. This design can be further expanded by introducing distinct adapter indices during ligation, allowing hundreds of samples to be pooled and sequenced together in a single run. Moreover, because samples are individually labelled during the initial PCR, the approach reduces the risk of cross‐contamination and handling errors commonly associated with two‐step PCR workflows. Despite its advantages, the in‐line PCR strategy remains less commonly employed in the literature than the two‐step PCR method. Careful evaluation is required to determine whether custom‐designed barcodes interfere with target‐specific primer binding during PCR, as this could introduce biases in microbial community profiling.

**FIGURE 1:**
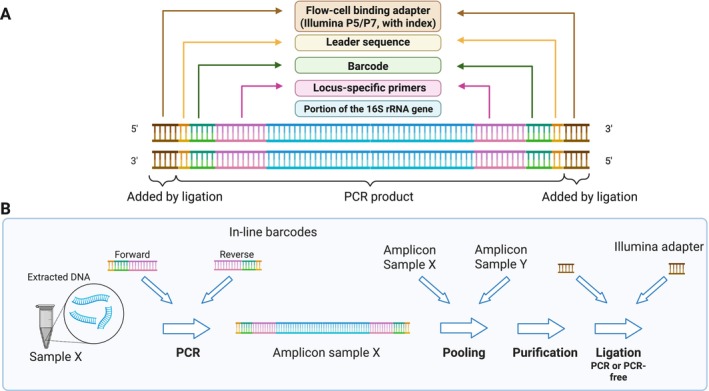
Structure of the final sequencing libraries and overview of experimental workflow. (A) Composition of the final sequencing libraries after PCR amplification and adapter ligation, showing the arrangement of primer, barcode, and adapter regions. (B) Flowchart of the one‐step in‐line barcoding workflow used for 16S rRNA gene metabarcoding. Created with BioRender.

In this study, we developed and evaluated an in‐line PCR protocol for 16S rRNA gene amplicon sequencing tailored to microbiome research. We designed and tested 136 bacterial and 156 archaeal in‐line barcoded primer pairs on three anaerobic digestion enrichment samples and a mock gut microbiome community. These primers incorporated a universal 16S rRNA gene primer sequence, a library barcode from an established workflow (Nextera XT, Illumina), and a short “leader sequence”, here defined as a neutral sequence placed upstream of the barcode to minimize barcode‐dependent effect on adapter ligation (Figure 1A). We also compared PCR‐free and PCR‐based adapter ligation strategies. Specifically, we (1) assessed barcode fidelity, recovery rates, and mismatch profiles, (2) examined the impact of barcode identity on microbial community composition, and (3) identified two optimized sets of 96 barcoded primer pairs for bacterial and archaeal communities that demonstrated the highest robustness and reproducibility across heterogeneous samples. To support users, we present an end‐to‐end workflow for microbial community profiling that combines an experimental protocol with practical guidelines, key parameters, and troubleshooting tips, and a bioinformatics pipeline for demultiplexing, quality control, and taxonomic inference using validated, widely adopted tools. This streamlined approach offers a simple, scalable, and cost‐effective alternative to conventional multi‐step methods while ensuring reproducibility.

## 2 Materials and Methods

### 2.1 Design of Barcoded Primers

To develop and evaluate a scalable in‐line barcoding workflow for microbiome profiling, we designed two sets of primers, each comprising three elements (Figure 1 and Table S1): a universal 16S rRNA gene primer, an 8‐bp Illumina barcode from the Nextera XT index set (*Oligonucleotide sequences 2020 Illumina Inc. All rights reserved*), and a 5‐bp leader sequence. Unlike heterogeneity‐spacer or staggered primers, which can introduce annealing bias (Jensen et al. 2019; Tremblay et al. 2015), our design omitted spacers to minimize amplification variability and simplify library preparation. Similarly, the leader sequence is meant to limit the influence of the barcode sequence on adapter ligation efficiency (map generation on barcode sequences). For bacteria, we used the 338F/785R primer pair targeting the V3‐V4 region (amplicon≈447 bp) (Abellan‐Schneyder et al. 2021; Albertsen et al. 2015; Apprill et al. 2015; Caporaso et al. 2011; Fadeev et al. 2021; Mazzoli et al. 2020; Parada et al. 2016; Walters et al. 2015), and for archaea, the 519F/915R pair targeting the V4‐V5 region (amplicon≈397 bp) (Albertsen et al. 2015; Pausan et al. 2019; Roth et al. 2023; Wei et al. 2019). Both sets are widely used for community profiling (Abellan‐Schneyder et al. 2021; Pausan et al. 2019) and are compatible with 250–300 bp paired‐end sequencing, ensuring sufficient overlap for accurate assembly. In total, 136 bacterial (8 forward × 17 reverse) and 156 archaeal (13 forward × 12 reverse) primer‐barcode combinations were tested. Because primer binding regions are partly conserved across domains (Baker et al. 2003; Klindworth et al. 2013; Takahashi et al. 2014; Walters et al. 2015), some cross‐domain amplification was expected and accounted for in downstream analyses. Detailed thermodynamic properties of all primers have been calculated using the OligoAnalyzertool (Integrated DNA Technologies, USA) and are provided in Table S2.

### 2.2 Experimental Workflow

The workflow was evaluated using five independent enrichment cultures derived from anaerobic digestion sludge collected from a municipal wastewater treatment plant in Vaud, Switzerland (46°36′N, 06°32′E). These cultures, enriched under different conditions and containing both bacteria and archaea, were selected to ensure the robustness of the primer evaluation by providing different biological inputs. In addition, a ZymoBIOMICS Gut Microbiome Standard (Zymo Research, cat. no. D6331, USA), which comprises 21 microbial strains, including two fungi and one archaeon, was also included. Bacterial primer sets were tested on two enrichment cultures and the Zymo mock community, while archaeal primer sets were tested on three enrichment cultures. For each sample, all primer‐barcode combinations were tested (156 for archaeal and 136 for bacterial primer sets), allowing systematic assessment of primer performance and barcode‐associated variability under identical conditions and processes. After amplification with barcoded primers, we evaluated different ligation strategies, comparing PCR‐free versus PCR‐plus workflows. In addition, we later tested an equivolume pooling approach (using the mock community) as a practical alternative to equimolar pooling of all amplicons. A schematic overview of the experimental design is shown in Figure 1.

### 2.3 Laboratory Methods

#### 2.3.1 DNA Extraction

DNA was extracted using the DNeasy PowerSoil Pro Kit on a QIAcube automated platform (Qiagen, USA), following the manufacturer's protocol. Prior to extraction, samples were pre‐heated at 55°C for 5 min in CD1/EB buffer, then bead‐beaten (Precellys, Bertin Instruments, France; 5500 rpm, 2 × 30 s, 30 s cooling). Lysates were centrifuged (12,000 *g*, 1 min), and 600 μL of supernatant was processed for automated purification. DNA was eluted in 100 μL of C6 buffer, quantified by NanoDrop (Thermo Fisher Scientific, USA), and stored at −80°C. The same procedure was applied to both environmental samples and the ZymoBIOMICS mock community.

#### 2.3.2 PCR Amplification

PCR amplifications were performed using MyFi 2× DNA Polymerase Mastermix (Meridian Bioscience, UK) following the manufacturer's recommendations. Primers (Integrated DNA Technologies, USA) were synthesized with standard desalting purification and resuspended in molecular‐grade water. DNA templates were normalized to 0.1 ng μL^−1^, and PCRs were run for 30 cycles to simulate conditions typical of low‐input samples and to accentuate potential amplification biases. The temperature ramp rate between denaturation and annealing was reduced to 1.6°C s^−1^ to enhance primer‐template alignment. Detailed cycling conditions are provided in Table 1.

**TABLE 1:** PCR cycling protocol for bacterial and archaeal barcoded primers.

Step	Temperature (°C)	Time (s)	Cycles
Initial denaturation	95	60	1
Phase 1			10
Denaturation	95	15	
Annealing (bacteria/archaea)	56/53	15	
Extension	72	15	
Phase 2			20
Denaturation	95	15	
Annealing	60	15	
Extension	72	15	
Final extension	72	45	1

#### 2.3.3 Library Preparation

PCR products were purified using SPRIselect beads (0.8× ratio, Beckman Coulter) and eluted in 30 μL of nuclease‐free water. DNA concentrations were measured with the Quant‐iT PicoGreen assay (Life Technologies) before equimolar pooling using a Myra liquid‐handling robot (Biomolecular Systems, Australia). Libraries were constructed with the xGen DNA Library Prep MC Kit (IDT, USA), following the manufacturer's instructions. For the PCR‐free workflow, full length indexed adaptors (TruSeq DNA UD Indexes v2, Illumina, USA) were ligated to 100 ng input DNA. For the PCR‐plus workflow, stuby adaptors (IDT, USA) were ligated to 50 ng input DNA, and amplification was performed for 5 cycles with xGen UDI 10 nt indexing primers (IDT, USA). Final libraries were purified (0.8× beads), quantified by Qubit fluorometry, and checked for fragment size on a Fragment Analyser (Agilent, USA).

For high‐throughput testing, we followed an equivolume pooling strategy. Unpurified amplicons (5 μL each) were pooled and the pool was purified at a 0.8× beads ratio as described above. Sequencing libraries were made following the PCR‐free protocol.

#### 2.3.4 Sequencing of 16S rRNA Amplicon Libraries

Sequencing was carried out on an Element Biosciences Aviti system using the Cloudbreak FS Standard Output 600‐cycle kit, run in paired‐end mode (2 × 300 cycles). Polony map generation was set on the 16S rRNA gene primer barcode sequence at the beginning of read 1 (high diversity region). PhiX was spiked at approximately 1%. Base calling and demultiplexing were performed using *bases2fastq* (v2.0.0.1379264253).

#### 2.3.5 Whole Genome Library Preparation and Sequencing

The ZymoBIOMICS Gut Microbiome Standard (Zymo Research, cat. no. D6331, USA) was used for whole‐genome sequencing and assessment of species composition. A total of 150 ng of input DNA was used to construct the library using the Illumina DNA Prep kit (Illumina, USA), incorporating unique dual‐indexed adapters (Illumina). Libraries were PCR‐amplified for 5 cycles, first purified as recommended by the manufacturer, further purified at a stringent 0.65× bead ratio, quantified using a Qubit fluorometer (Life Technologies, USA), and evaluated for fragment size distribution using a Fragment Analyser (Agilent Technologies, USA). Sequencing was performed on the Aviti system using the Cloudbreak FS High Output kit (2 × 150 bp). Base calling and demultiplexing were conducted with *bases2fastq* (v2.0.0.1379264253).

### 2.4 Bioinformatics and Data Processing

#### 2.4.1 Demultiplexing and Quality Filtering

All demultiplexing and trimming steps were performed in a Unix‐based environment (WSL, Python 3.12.8) using Cutadapt v4.9 (Martin 2011). Adapter sequences (leader 5′‐GCATC) were removed in paired‐end mode with zero‐mismatch tolerance, followed by demultiplexing with a one‐mismatch threshold across the full 8‐bp barcode and a minimum 8‐bp overlap. Forward and reverse reads were assigned to barcode pairs using a custom FASTA barcode list (Data S1), and primers were subsequently trimmed. Processing scripts are available on GitHub (Lisajrd/16‐pipeline). Reads were then processed following the DADA2 workflow (Callahan et al. 2016), including quality filtering (maxEE = 2, truncQ = 2, maxN = 0), independent error modelling for forward and reverse reads, and merging with *mergePairs()*. Read quality was assessed with FastQC. A representative quality profile is provided in (Figure S1).

#### 2.4.2 Chimera Detection and Taxonomic Affiliation

Following quality filtering and denoising, ASV tables were generated using the *makeSequenceTable()* (DADA2). Bacterial reads were restricted to 400–500 bp, while archaeal sequences were retained within a 350–500 bp range. These size thresholds correspond to the expected amplicon sizes from the primer sets and include slightly expanded margins to account for natural length variation within the 16S rRNA gene (Vargas‐Albores et al. 2017; Yarza et al. 2014). Chimeras were removed with *removeBimeraDenovo()* (consensus method, default parameters). Taxonomic assignment was performed against the SILVA v138.1 database trained for species‐level classification using *assignTaxonomy()*. Final ASV, taxonomy, and metadata tables were compiled into phyloseq objects (McMurdie and Holmes 2013) for downstream statistical analyses and visualization.

#### 2.4.3 Rarefaction

Before diversity analyses, sequencing depth was standardized by rarefaction using rarefy_even_depth() in phyloseq with a fixed random seed. Although the use of rarefaction remains debated (McKnight et al. 2019; McMurdie and Holmes 2013; Schloss 2024; Weiss et al. 2017), recent evaluations support its effectiveness in controlling for uneven sequencing effort in amplicon datasets while preserving the ecological validity of diversity and ordination metrics (Schloss 2024). It is crucial, however, to ensure that the subsampling depth used in rarefaction preserves sufficient coverage to capture community composition reliably. This is typically assessed using rarefaction curves, which are presented in Figure S2. Rarefaction was performed using the *rarefy_even_depth()* (phyloseq) with a fixed random seed for reproducibility. Rarefaction depths corresponded to the minimum sample read counts post‐filtering: Archaea_1 (PCR, 10,669; PCR‐free, 12,342), Archaea_2 (PCR, 17,826; PCR‐free, 32,687), Archaea_3 (PCR, 13,147; PCR‐free, 21,961), Bacteria_1 (PCR, 21,389; PCR‐free, 26,288), Bacteria_2 (PCR, 19,898; PCR‐free, 27,441), Zymo (PCR, 60,639; PCR‐free, 71,550).

#### 2.4.4 Alpha Diversity Indexes

Alpha diversity was evaluated across barcode pairs using the Hill numbers framework to provide comparable and complementary measures in the common unit *of* effective number of taxa (Alberdi and Gilbert 2019; Hill 1973). We report *q* = 0 (richness (Gotelli and Colwell 2001)), *q* = 1 (exp(Shannon), reflecting the effective number of abundant taxa), and *q* = 2 (1/Simpson), emphasizing dominant taxa (Kim et al. 2017). For interpretability and completeness, we additionally computed Pielou's evenness (J) (Pielou 1966) and Hill‐based evenness derived as the ratio of diversity of order *q* to species richness (Eq = q1/q0 and q2/q0 (Chao and Ricotta 2019)). Analyses were performed at the ASV level for archaeal libraries and at the species level for bacterial libraries, reflecting differences in taxonomic resolution between datasets. Evenness is particularly informative in this context, as consistent values across primer‐barcode combinations indicate preserved relative abundances and minimal amplification bias (Ehsani et al. 2018; Wittebolle et al. 2009; Zhang et al. 2021). Together, these indices provided a robust evaluation of barcode effects on taxon detection and abundance patterns (Ashton et al. 2016; Regueira‐Iglesias et al. 2023).

#### 2.4.5 Differential Abundance Analysis

Differential abundance testing was performed using ANCOM‐BC2 (Lin and Peddada 2024), with barcode combination as the fixed effect. Pairwise comparisons were conducted with FDR correction (*α* = 0.05). Analyses accounted for compositional data structure and structural zeros and were performed on non‐rarefied datasets.

#### 2.4.6 Analysis of Metagenomic Data

Raw paired‐end metagenomic reads were processed with the nf‐core/mag pipeline (Ewels et al. 2020) (v5.0.0) under Nextflow (v24.10.0) on the SCITAS HPC cluster (EPFL) using Singularity containers. Reads were quality‐filtered and trimmed with fastp (Chen et al. 2018), assembled with MEGAHIT (Li et al. 2015), and mapped back to contigs using Bowtie2 (Langmead and Salzberg 2012). Metagenome‐assembled genomes (MAGs) were reconstructed via differential coverage binning with MetaBAT2 (Kang et al. 2019) and MaxBin2 (Wu et al. 2016), refined with DAS Tool (Sieber et al. 2018), and assessed for completeness and contamination using CheckM2 (Chklovski et al. 2023). Taxonomic assignment was performed with GTDB‐Tk (Chaumeil et al. 2020) against the GTDB r226 database.

## 3 Results

### 3.1 Comparison of PCR Yields

As an initial proxy for evaluating consistency among barcoded primer pairs, we compared PCR yields and inspected amplicons by agarose gel electrophoresis. A representative gel image is provided in Figure S3A. Individual purification of amplicons was performed to precisely quantify their concentrations and evaluate the impact of barcode sequences on PCR yield. PCR yield and reproducibility were then assessed on both mock and environmental DNA samples (Figure S3B). Raw yield distributions revealed that Archaea_1, Archaea_2, and Bacteria_1 samples exhibited narrow, unimodal patterns indicative of consistent amplification, whereas other sets showed broader distributions, suggesting higher variability. Overall yields were stable, averaging around 30 ng μL^−1^, with Archaea_1 reaching slightly higher values (~40 ng μL^−1^). A Wilcoxon rank‐sum test confirmed modest but significant differences between archaeal and bacterial primer sets (35.0 ng μL^−1^ vs. 30.6 ng μL^−1^; *p* = 6.18 × 10^−4^), while variability within barcode combinations remained low. No significant pairwise differences were detected within primer sets after multiple‐testing correction (Kruskal–Wallis with Dunn's post hoc test).

### 3.2 Barcode Recovery and Read Distribution

#### 3.2.1 Demultiplexing Strategy

We next investigated barcode recovery and read distribution across demultiplexing strategies. Amplicons were pooled equimolarly, which should theoretically yield uniform read counts across barcode combinations. The primary goals were to (i) identify the more accurate method for assigning sequencing reads to their corresponding barcoded primer combinations, (ii) examine the position and integrity of barcode sequences within reads, including the presence of sequencing errors or indels, and (iii) evaluate read distribution among barcoded primers. Given the use of normalized inputs, we expected uniform barcode representation, with theoretical proportions calculated based on the number of in‐line barcode pairs used for bacterial and archaeal libraries.

**FIGURE 2:**
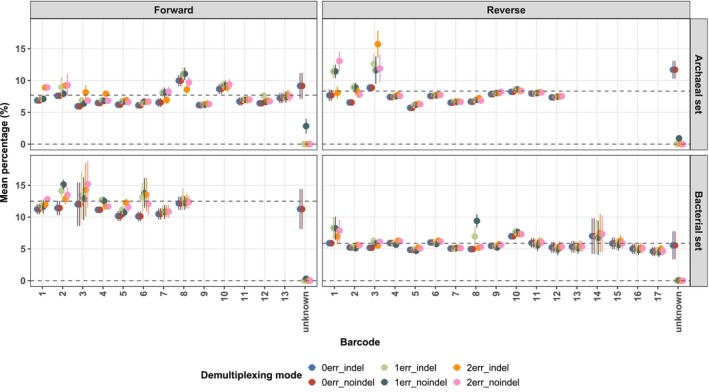
Theoretical and experimental mean percentages of barcodes. Mean theoretical and observed relative abundances of forward (“if”) and reverse (“ir”) barcodes are shown for archaeal and bacterial primer sets. Data are grouped by demultiplexing stringency: Exact matches (0err), one mismatch allowed (1err), and two mismatches allowed (2err), with or without indel tolerance. Dashed lines indicate expected theoretical abundances.

Demultiplexing accuracy was assessed by calculating the proportion of reads assigned to each barcode, normalizing barcode‐specific read counts to the total number of reads per mode and sample, including those with unassigned barcodes. For each barcode, the mean percentage and standard deviation were calculated across sample groups (e.g., archaeal or bacterial barcode sets). Observed barcode distributions were compared to theoretical expectations, assuming a uniform distribution. An additional category, ‘unknown’ barcodes, representing reads unassigned to any known barcode and expected at a theoretical frequency of 0%, was included. These results are illustrated in Figure 2, with the full dataset provided in Table S3.

Demultiplexing performance depended strongly on error tolerance. Stringent zero‐mismatch conditions caused a notable loss of reads classified as “unknown” (e.g., 9.15% ± 1.97% for forward archaeal tags), while allowing one substitution error greatly improved assignment rates across all groups. Primer‐specific patterns were also evident: *Forward_Archaea_*5 and *_6* consistently yielded lower recovery (~6.7%), whereas *Reverse_Archaea_8* slightly exceeded the theoretical rate (9.21% ± 0.73% vs. 8.33%). Some primers, such as *Forward_Bacteria_6*, showed higher variability (14.66% ± 2.52%), indicating inconsistent recovery among barcode combinations.

Archaeal primers generally produced narrower and more uniform read distributions than bacterial ones; a pattern maintained under relaxed error settings except for an overrepresentation of *Reverse_Archaea_3* (15.7% ± 2.03%). Among bacterial barcodes, variability was greatest for forward tags (especially 1, 3, 4, 5, and 8), while reverse tags remained relatively stable except for *Reverse_Bacteria_10*. Allowing one mismatch assigned 94% of reads to barcode pairs, increasing to 99% when indels were also permitted.

Based on these results, we adopted a demultiplexing threshold permitting one mismatch and one indel to maximize read retention while minimizing ambiguous sample assignments. This choice is consistent with current amplicon‐sequencing standards, which typically tolerate up to two mismatches per barcode.

#### 3.2.2 Barcode Recovery

Consistent with prior reasoning, read counts for valid barcode pairs were predicted to follow a uniform distribution, with theoretical proportions of 1/136 for bacterial and 1/156 for archaeal tag combinations. Barcode pairs containing an “unknown” tag were expected to yield zero reads. Observed read distributions were therefore compared to these theoretical expectations using the selected demultiplexing parameters (Figure S4).

For archaeal barcode pairs, read counts clustered tightly around expected values, indicating overall balanced recovery. Minor deviations were observed, notably for *Reverse_Archaea_3*, which showed a modest overrepresentation across combinations, and *the Forward_Archaea_8‐Reverse_Archaea_2* pair, which produced the highest outlier signal. For bacterial barcodes, most combinations showed low variability and agreement with theoretical proportions. Slightly elevated variability was observed for forward barcodes 1, 4, and 8 paired with reverse barcodes 13–17, while a few combinations (e.g., *Forward_Bacteria_2‐Reverse_Bacteria_1/8* and *Forward_Bacteria_3‐Reverse_Bacteria_14*) yielded higher‐than‐expected read proportions.

#### 3.2.3 Read Distribution and Retention During Downstream Processing

We evaluated sequencing yield and data retention following *in silico* amplicon processing under both PCR and PCR‐free protocols. For each sample, read counts were assessed across key processing steps, and distributions across barcoded primer pairs were compared. Detailed results are presented in the Supporting Informations (Figure S5). Among archaeal samples, the average number of filtered read counts across barcode pairs ranged from 60,551 ± 15,610 to 155,817 ± 42,640 reads, with final non‐chimeric read counts between 41,497 ± 17,303 and 121,165 ± 35,702. Archaeal libraries exhibited relatively consistent read count distributions across barcode pairs, with PCR‐free libraries generally yielding a higher percentage of recovery of filtered and non‐chimeric reads compared to PCR‐based libraries. Notably, samples Archaea_1 and Archaea_3 exhibited significant differences between PCR and PCR‐free groups (Wilcoxon rank‐sum test, *p* < 0.05). In contrast, bacterial and mock community samples exhibited greater variability. The number of average filtered read counts across barcode pairs ranged from 49,633 ± 14,486 to 144,680 ± 24,027, while non‐chimeric read retention followed a similar pattern (41,037 ± 42,230 to 107,270 ± 19,583). Consistent with the archaeal results, PCR‐free bacterial libraries generally exhibited higher and more consistent read recovery following chimera removal. Statistical analysis using Wilcoxon rank‐sum tests revealed a significant difference for sample *Bacteria_2* between PCR‐treated and PCR‐free libraries.

### 3.3 Community Composition and Diversity Metrics

#### 3.3.1 Taxonomic Profiling

**FIGURE 3:**
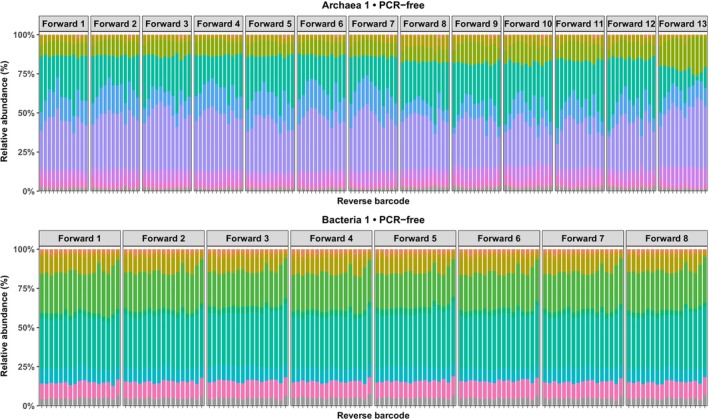
Comparison of microbial community profiles across barcode combinations in representative environmental samples *(Archaea_1 and Bacteria_1)* following in‐line barcoding and ligation‐based, PCR‐free, library preparation. *The top 15 taxa per dataset are shown for each barcode pair*. Panels correspond to forward barcodes; for readability, reverse barcodes are not shown in the legend. Reverse barcodes are numbered sequentially (1–8 for archaeal tags and 1–17 for bacterial tags). A legend for the taxa is provided in Figure S6.

The impact of barcoded primer design on microbial community profiling was assessed by comparing relative abundance patterns across all samples and barcode pair combinations, as illustrated in Figure 3 and further expanded in Supporting Informations (Figure S6). The archaeal primers were not entirely specific to Archaea, a well‐documented limitation consistent with previous reports (See part 2.1).

As shown in Figure 3, the sample Archaea_1 exhibited moderate variability among barcode combinations, with the greatest deviations observed when using the barcoded primer *Forward_Archaea_13*. Despite this variability, taxonomic distributions remained relatively even overall. In contrast, Archaea_3 (Figure S6), which was characterized by lower alpha diversity and strong dominance by a few taxa, primarily *Methanobrevibacter* and *Methanosphaera*, displayed a markedly less even distribution. This pattern suggests that barcode performance may be modulated by community complexity, taxonomic composition, or DNA quality. Overall, barcode profiles were largely consistent across archaeal samples, although *Forward_Archaea_13* repeatedly produced more distorted community structures compared to other forward primers, as well as *Reverse_Archaea_1* and *Reverse_Archaea_10*. In the more complex Bacteria_1 sample, barcode pairs generated broadly comparable community profiles, with dominant taxa consistently recovered and only minor fluctuations observed among subdominant groups (Figure 3).

**FIGURE 4:**
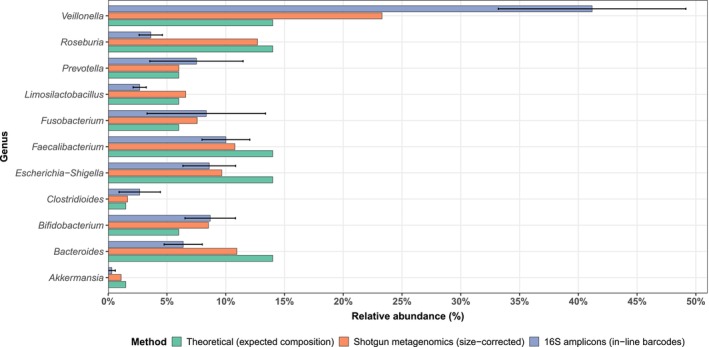
Comparison of relative genus‐level abundances in the mock community obtained from theoretical composition, metagenomic sequencing (shotgun, genome‐size corrected), and 16S rRNA gene sequencing (in‐line barcodes, copy‐number corrected).

To provide an independent reference, a shotgun metagenomic profile of the mock community was generated and compared to both the theoretical composition provided by the manufacturer and the averaged 16S rRNA profile obtained from in‐line barcodes (Figure 4). Overall, the shotgun‐derived abundances closely resembled the expected theoretical composition, with moderate deviations likely attributable to DNA extraction biases. For instance, *Escherichia‐Shigella* and *Faecalibacterium* tended to be underrepresented relative to theoretical values, whereas *Veillonella* appeared slightly enriched. 16S rRNA gene‐based profiling showed more pronounced discrepancies compared to the theoretical and shotgun‐derived compositions, notably an overrepresentation of *Veillonella rogosae* (≈40% versus≈25% in shotgun). As broadly documented for 16S rRNA‐based strategies (see Introduction), such discrepancies likely reflect amplification and primer‐associated biases.

Across different barcode combinations, relative abundances within the mock community were largely consistent, with only minor deviations for taxa such as *Enterococcus* and *Limosilactobacillus*. The overall low standard deviations among barcode pairs indicate limited barcode‐induced bias and confirm the high reproducibility of the in‐line barcoding approach. Furthermore, to evaluate whether barcode pair combinations introduced taxon‐specific biases, we assessed the relative representation of individual taxa across all combinations for both bacterial and archaeal datasets. Using ANCOMBC2 at the genus level, we detected no significantly over‐ or underrepresented taxa across barcode combinations, indicating that the choice of barcode pairs did not introduce systematic taxonomic bias.

#### 3.3.2 Diversity Indexes

##### 3.3.2.1 Intra‐Sample Diversity

**FIGURE 5:**
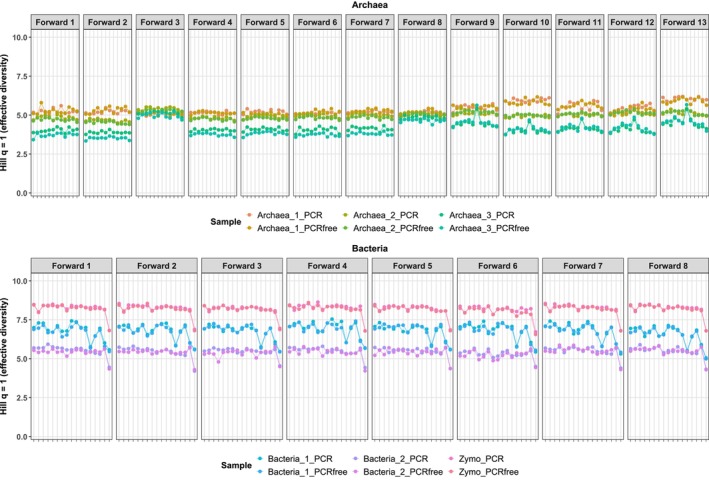
Effective alpha diversity (Hill *q* = 1) for archaeal (top) and bacterial (bottom) datasets. Panels correspond to forward barcodes; for readability, reverse barcodes are not shown in the legend. Reverse barcodes are numbered sequentially (1–8 for archaeal tags and 1–17 for bacterial tags).

Alpha diversity metrics were calculated for each barcode combination across library preparation methods (PCR vs. PCR‐free) and sample (Figure S5). In bacterial samples and the mock community, diversity estimates were highly consistent between PCR and PCR‐free libraries, with minimal variation observed among barcode combinations. Standard deviations remained low across all metrics (e.g., Hill *q* = 1: ±0.31–0.53; Hill *q* = 2: ±0.32–0.58), indicating robust reproducibility. Within each seed culture, barcode‐specific diversity values were closely aligned. In contrast, archaeal samples exhibited more pronounced effects of PCR amplification. While inter‐barcode variability remained limited for Hill indices, significant differences were detected between PCR and PCR‐free libraries across all archaeal seed cultures. For example, Hill *q* = 2 (Archaea_2: *p* = 1.4 × 10^−14^, Wilcoxon signed‐rank test) and observed richness (Hill *q* = 0; Archaea_2: *p* = 1.7 × 10^−23^) differed markedly by library type, with PCR‐based libraries yielding higher diversity. These shifts were accompanied by moderate differences in standard deviation across barcodes, particularly for richness (e.g., Archaea_3: PCR‐free ±3.10 vs. PCR ±1.90; Archaea_1: PCR‐free ±1.33 vs. PCR ±1.61). Across all samples, observed richness showed greater variability than abundance‐weighted metrics, with standard deviations exceeding 10% of the mean in all cases except for the mock community. This pattern reflects the inherent greater sensitivity of richness to rare taxa, compared to Hill *q* = 1 and *q* = 2 indices. Detailed diversity values for each barcode combination and sample are shown in Figure 5, using the Hill *q* = 1 index as an example. The full dataset is available in Figure S5.

In archaeal samples, alpha diversity remained broadly stable across barcode combinations; only *Forward_Archaea_3*, *_8*, and _*9* produced slightly higher values than the other primer pairs (Archaea_1, no PCR, Hill *q* = 1, *p* = 6.1 × 10^−20^, ANOVA). Differences between PCR and PCR‐free conditions were otherwise limited, except for sample Archaea_3, which had the lowest overall diversity (Hill *q* = 1≈4), compared to higher values in Archaea_1 and Archaea_2 (≈5.25). For bacterial samples, Hill *q* = 1 was largely consistent across barcode combinations, and PCR vs. PCR‐free differences were small with overlapping values, except for a slight divergence in *Bacteria_2*. Certain barcode combinations, however, exhibited systematic effects on diversity: Reverse_Bacteria_17 was consistently associated with lower values (a decrease of more than 10% in Bacteria_2 relative to the mean of the other pairs), and *Reverse_Bacteria_13* showed a similar but weaker trend. Overall, the reverse barcoded primer significantly affected diversity estimates across bacterial datasets and most diversity metrics (e.g., Bacteria_1 PCR, Hill *q* = 1, *p* = 0.008, ANOVA).

##### 3.3.2.2 Extending Diversity Analysis: Assessing Beta Diversity Across Primer‐Barcoded Libraries

**FIGURE 6:**
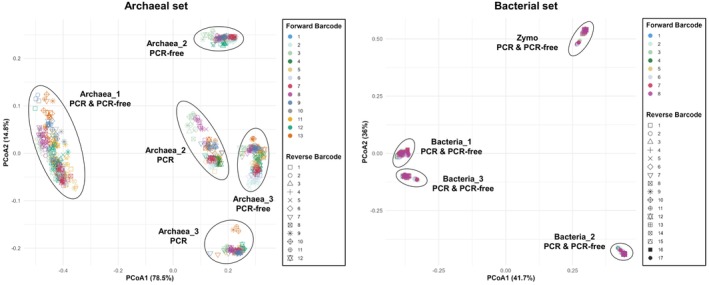
Principal coordinates analysis of archaeal (left) and bacterial (right) community composition based on Bray‐Curtis distances. Ellipses represent samples.

We further examined beta diversity, assessing variation between samples relative to within‐sample diversity with principal coordinates analysis (PCoA) based on Bray‐Curtis distances. In archaeal samples (Figure 6A), inter‐sample variation exceeded intra‐sample variation. PCR and PCR‐free libraries for *Archaea_2* and *Archaea_3* clustered separately, but this separation was mainly along the second axis, which explained a small fraction of variance and therefore had limited interpretive relevance. For the bacterial set (Figure 6B), all libraries derived from the same sample clustered tightly and overlapped, irrespective of the library preparation protocol (PCR or PCR‐free) or the barcoded barcode pair. After verifying the homogeneity of multivariate dispersions (betadisper test, *p* > 0.05), a PERMANOVA test performed on the Bray‐Curtis distances confirmed that the choice of barcoded primer pair did not significantly influence overall community composition.

### 3.4 Selection of 96 Barcoded Primer Pairs for Multiplexed Amplicon Sequencing

Out of the 156 and 136 in‐line barcode pairs evaluated for profiling archaeal and bacterial communities, respectively, we aimed to identify 96 barcode pairs with consistently robust performance, enabling compatibility with standard 96‐well plate formats for high‐throughput amplicon library preparation. Barcode pair performance was previously quantitatively assessed using multiple complementary metrics, including PCR amplification yield, alpha diversity indices, and community evenness. A Bray–Curtis dissimilarity‐based metric was developed to directly quantify the influence of barcode pairs on community composition within the same sample.

For each sample, one barcode pair was designated randomly as a reference, and pairwise Bray‐Curtis distances were calculated relative to this reference to capture deviations in taxonomic profiles. To identify barcode pairs that performed similarly overall, a principal component analysis (PCA) was performed on the bacterial and archaeal barcode datasets separately, using the combined set of performance metrics. The first three principal components were retained, together explaining over 90% of the total variance in each dataset. Barcode pairs were then clustered using *k*‐means clustering in this multivariate space to group those with closely aligned performance profiles. The consistency of barcode pair behaviour across samples was further evaluated by computing co‐clustering frequencies, defined as the proportion of samples in which any two barcode pairs were assigned to the same *k*‐means cluster. These frequencies were aggregated into domain‐specific co‐clustering matrices, which were then converted into dissimilarity matrices (1—co‐clustering frequency). Hierarchical clustering of these matrices enabled the identification of higher‐order “metaclusters” of barcode pairs exhibiting consistently similar performance across diverse sample types (Figures S7 and S8).

**FIGURE 7:**
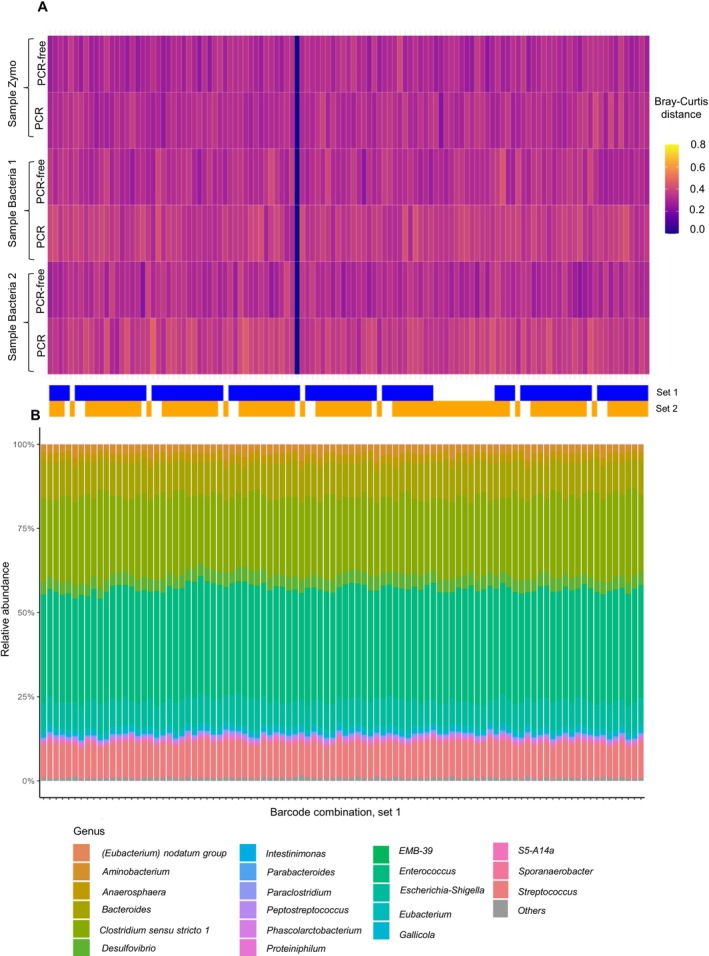
Bacterial sample diversity and taxonomic profiles across selected barcode combinations. (A) Bray–Curtis dissimilarity to a randomly selected reference barcode pair (highlighted in blue) across selected barcode combinations for all bacterial samples. Barcode combinations are colour‐annotated based on their inclusion in barcode sets 1 (blue) and 2 (orange). (B) Taxonomic composition of the *Bacteria_1_PCRfree* sample across the same set of barcode combinations.

In selecting the final barcode pair, we prioritized minimizing the number of unique forward and reverse primers in order to streamline library preparation and reduce complexity without compromising performance. This strategy provided an effective balance between technical simplicity and profiling consistency. For the archaeal set, 96 barcode pairs were retained, consisting of forward primers 1–12 and reverse primers 3–8 and 10–11. A similar selection process was applied to the bacterial barcoded primer set. The combination demonstrating the most robust performance across all tested samples, hereafter referred to as Set 1, included forward primers 1–5 and 7–8, paired with reverse primers 1, 3–7, 9–12, and 14–15, along with additional combinations involving forward primers 1–5 and 7 with reverse primers 2 and 8. However, to facilitate broader adoption, we also propose a simplified bacterial barcode set (Set 2), comprising forward primers 1–8 and reverse primers 1–12 that exhibited consistently high performance. Minor impacts were observed on relative taxonomic abundances in Bacteria_2, but alpha diversity metrics remained stable, indicating no effect on overall community diversity. This streamlined set represents a practical and reliable option for routine applications, balancing usability with robust and reproducible profiling. A complete list of selected barcode pairs with the most consistent results is provided in Table S6. Selection outcomes for the bacterial and archaeal sets are illustrated in Figure 7 and Figure S9, respectively.

### 3.5 Exploring the Role of Additional Factors in Library Preparation

#### 3.5.1 Effect of PCR After Adapter Ligation

As discussed previously, the tagged PCR may introduce artefacts, such as tag‐jumping, when a final PCR amplification is performed after adaptor ligation (Bohmann et al. 2022; Carøe and Bohmann 2020). To assess the impact of omitting this step, we compared PCR‐free and PCR‐based library preparation protocols across archaeal, bacterial, and mock community libraries. PCR‐free preparations consistently yielded higher numbers of filtered, non‐chimeric reads, with reduced variability across barcode pairs (Figure S5). This improvement was particularly marked in archaeal samples, where low‐diversity communities were more susceptible to PCR‐induced biases affecting the percentage of reads recovered after chimera removal and alpha diversity metrics (Figure 6 and Table S5). While bacterial samples were less strongly affected, PCR‐free libraries still showed modest improvements in read retention and consistency. Beta diversity analyses further confirmed that inter‐sample biological variation exceeded the technical variation introduced by the library preparation method or barcode selection (Figure 6).

#### 3.5.2 Effect of Pooling Strategy

We compared two pooling strategies to test their impact on community profiles: the standard equimolar approach and a simplified equivolume pooling followed by a single purification step (Figure 1). The latter, applied to mock communities with narrow DNA concentration ranges (28.4 ± 7.4 ng μL^−1^; Figure 2), substantially reduces handling time and cost. Diversity metrics were compared between pooling methods using paired *t*‐tests, which showed no significant difference across all metrics. Consistently, genus‐level profiles and differential abundances, evaluated with ANCOM‐BC2 using the pooling method as a fixed factor, detected no significant taxa‐level shift (Figure S7).

## 4 Discussion and Conclusion

This study validates a one‐step in‐line barcoding strategy for 16S rRNA gene amplicon sequencing. We systematically assessed the impact of barcode design and sequencing library preparation on taxonomic representation, diversity metrics, and community composition profiling by evaluating 136 and 156 barcoded primer pairs across mock and complex bacterial and archaeal communities.

Overall, PCR amplification using normalized DNA inputs yielded reproducible results, with most barcoded primers supporting uniform read output and high library complexity. Non‐significant differences were observed in amplification efficiency among certain primer sets, which did not compromise overall taxonomic profiles. PCR‐free ligation protocols may reduce technical biases by limiting chimera formation and other PCR‐derived artefacts, particularly in low‐biomass or taxonomically restricted samples. In our dataset, archaeal libraries prepared without post‐ligation PCR showed higher read recovery after chimera removal, supporting previous recommendations favouring PCR‐free approaches when feasible (Bohmann et al. 2022; Carøe and Bohmann 2020). Nevertheless, PCR‐based methods remain appropriate for low‐input samples, provided rigorous quality controls are in place. It was also demonstrated that equivolume pooling of PCR products performs comparably to the traditional equimolar pooling strategy when the samples fall in relatively narrow DNA concentration ranges. The former is a practical alternative for high‐throughput workflows. A semi‐quantitative gel‐based pooling adjustment method is proposed (detailed in Data S1), which enables volume corrections based on band intensity classification without requiring precise quantification.

Importantly, only primer pairs that consistently exhibited highly similar performance across a range of heterogeneous samples were retained in the final set for future applications, thereby ensuring robust and reproducible results in diverse microbial community contexts. We selected a specific set of 16S rRNA gene‐binding primers (see Section 2.1) due to their extensive use in comparative microbiome studies and their broad coverage of bacteria and archaea. Nonetheless, other primer sets targeting different regions could be readily integrated into this workflow, provided that equivalent validation is performed, as primer choice can strongly affect amplification efficiency and taxonomic recovery (Green et al. 2015; Kebschull and Zador 2015; Piñol et al. 2015; Sipos et al. 2007). More broadly, the workflow is compatible with any amplicon‐based sequencing project, including standard metabarcoding markers (e.g., ITS for fungi, 18S rRNA for eukaryotes, COI for animals, rbcL for plants) and targeted sequencing of functional genes. The inline‐barcode demultiplexing tool developed here is also fully generic and can be used independently of 16S rRNA gene analysis.

Across these selected barcode combinations, no systematic taxonomic biases were detected: dominant taxa were reliably recovered, and while minor variations in read abundance and alpha diversity were observed in certain barcode pairs, particularly in low‐diversity samples, these differences did not alter overall community structure or beta diversity patterns. Furthermore, attempts to correlate barcode properties (e.g., GC content, melting temperature, predicted secondary structure) with sequencing performance did not reveal any significant trends, suggesting the necessity of empirical validation when designing new barcode sets or implementing new workflows and indexing. We emphasize the need for careful bias assessment in 16S studies. Despite its widespread use, many microbiome studies neglect to test barcode‐ or primer‐induced biases. We strongly recommend preliminary validation on mock communities or sample subsets to ensure that labeling strategies do not introduce systematic distortions. We provide a comprehensive framework for such purposes, where all barcode combinations were explicitly tested on identical samples to confirm the absence of distortions, an approach rarely detailed in the literature. Metrics such as PCR yield, diversity indices, and Bray‐Curtis distances proved effective for evaluating primer performance and should be routinely included in future assessments. In general, caution is warranted when interpreting 16S rRNA gene sequencing data for community profiling, particularly when comparing relative abundances across samples or methodological approaches. The quantitative accuracy of 16S‐based approaches is still debated (Lamb et al. 2019), and analyses outcomes strongly depend on library preparation choices and analytical parameters (see Introduction), and likely deviate from those obtained with shotgun‐based methods (see Section 3.3.1).

From a practical standpoint, omitting the second amplification step reduces processing time and polymerase consumption. In addition, the short‐barcoded primers lower synthesis costs and can be used for multiple projects from a single synthesis batch, thereby amortizing the initial investment. Cost saving in purification depends largely on the pooling strategy (equivolume vs. equimolar), but the simplified workflow regardless reduces the steps of manipulations and associated labor time. Overall, this method offers a flexible and scalable alternative to commercial indexing kits, enabling cost‐effective implementation in large ecological and biomonitoring studies.

In summary, we present two sets of 96 in‐line barcoded primer pairs designed for both bacterial and archaeal community profiling, enabling scalable, cost‐effective, and reproducible microbiome analyses. A streamlined protocol incorporating volume‐based pooling and PCR‐free ligation has been validated, reducing contamination risks, minimizing handling errors, and eliminating some labor‐intensive, error‐prone, and costly steps of conventional workflows. Comprehensive testing across sample types demonstrated that this approach provides reliable performance. This approach, used with a standardized analytical framework, is suitable for large‐scale ecological applications, where high‐resolution taxonomic data must be balanced with practical constraints. Looking ahead, advances in barcode design, pooling strategies, and primer optimization, potentially supported by computational tools, will further improve reproducibility and scalability. Moreover, emerging technologies such as icon‐PCR (N6tec), designed to mitigate overamplification of dominant taxa (Jouvenot et al. 2024), offer promising avenues for reducing residual biases and enhancing the accuracy of community profiling.

## Author Contributions

L.J., J.M., J.W., and P.R. conceived and designed the study, including the development of barcoded primers. W.G. contributed to the study design and supervised the project. L.J. performed the library preparation, processed the experimental data, conducted the analyses, drafted the manuscript, and prepared the figures. A.C. and E.C. carried out quality checks, library purification, ligation, and sequencing. L.J. also established the GitHub repository and developed all bioinformatic processing scripts. V.P. participated in the elaboration of the demultiplexing pipeline. L.J., J.M., J.W., P.R., and W.G. contributed to data interpretation and manuscript revision. All authors discussed the results and provided critical feedback on the manuscript.

## Funding

This work was supported by swiss national science fundation, 200021_219222.

## Conflicts of Interest

The authors declare no conflicts of interest.

## Supporting information


**Figure S1:** Representative quality profile (Sample Zymo, ligation with PCR cycles) of paired‐end reads obtained from the in‐line barcoding workflow.
**Figure S2:** Rarefaction curves showing observed species richness as a function of sequencing depth for all samples.
**Figure S3:** Assessment of PCR yield and amplification consistency across tagged primer pairs. (A) Distribution of DNA concentrations measured by PicoGreen assays for bacterial and archaeal samples. Violin plots show the density of concentration values (ng μL^−1^), with white diamonds indicating mean values and black bars representing ±1 SD. (B) Representative agarose gel electrophoresis of amplicons from the Archaea_2 sample, amplified with 32 distinct tagged primer combinations: 4 forward barcodes (Forward_Archaea_1–4) each crossed with the same 8 reverse barcodes (Reverse_Archaea_1–8). Lane mapping: 1–8 = Forward_Archaea_1 × Reverse_Archaea_1–8; 9–16 = Forward_Archaea_2 × Reverse_Archaea_1–8; 17–24 = Forward_Archaea_3 × Reverse_Archaea_1–8; 25–32 = Forward_Archaea_4 × Reverse_Archaea_1–8. All reactions yielded single, sharp bands of the expected size (~400 bp). Gels were run in 0.5× TAE buffer on 1% agarose. NC denotes the negative control (PCR run without DNA template). Molecular weight marker: 1 Kb DNA Ladder RTU (GeneDireX Inc.).
**Figure S4:** Normalized read ratios per barcode pair for bacterial and archaeal samples in the “1‐error indel” mode. Dashed red horizontal lines indicate the theoretically expected proportions (1/136 for bacteria, 1/156 for archaea). To account for the fact that only a subset of the full barcode set was used in each sequencing run, read ratios were corrected using a factor reflecting the proportion of barcode combinations tested. Barcode pairs have been replaced by numerical identifiers; a correspondence table is provided in Table S4.
**Figure S5:** Read retention across processing steps. Dot plot showing the number of reads retained per library at each step of the processing pipeline.
**Figure S6:** Relative abundance barplots comparing microbial community composition across barcode combinations in all samples. Barcode pairs have been replaced by numerical identifiers; a correspondence table is provided in Table S4.
**Figure S7:** Hierarchical metaclustering of bacterial barcode pairs based on performance similarity across samples. Dendrogram showing hierarchical clustering (average linkage) of co‐clustering frequencies among 136 bacterial barcode pairs. For each sample, performance metrics (diversity metrics and Bray‐Curtis distances to a reference barcode pair) were reduced using PCA, and barcode pairs were clustered using k‐means (*k* = 2) in the resulting principal component space. Co‐clustering frequencies represent the proportion of samples in which each pair is grouped and were aggregated into a domain‐specific matrix. This matrix was transformed into a dissimilarity matrix (1—co‐clustering frequency) and clustered using *hclust* in R.
**Figure S8:** Hierarchical metaclustering of archaeal barcode pairs based on performance similarity across samples. Dendrogram showing hierarchical clustering (average linkage) of co‐clustering frequencies among 136 bacterial barcode pairs. For each sample, performance metrics (diversity metrics and Bray‐Curtis distances to a reference barcode pair) were reduced using PCA, and barcode pairs were clustered using *k*‐means (*k* = 2) in the resulting principal component space. Co‐clustering frequencies represent the proportion of samples in which each pair is grouped and were aggregated into a domain‐specific matrix. This matrix was transformed into a dissimilarity matrix (1—co‐clustering frequency) and clustered using *hclust* in R.
**Figure S9:** Comparison of species‐level relative abundance across barcode combinations in individual and pooled library preparations.
**Figure S10:** Archaeal profiles with selected tagged barcode pairs. (A) Bray‐Curtis distance to a randomly selected barcode pair across the selected barcode combinations for all bacterial samples. (B) Taxonomic profile of sample Archaea 3 across the selected barcode pairs using the PCR‐free ligation protocol.
**Table S1:** Sequences of custom‐designed tagged primers for 16S rRNA gene sequencing. Each primer includes a unique barcode tag, the leader sequence (underlined), and a 16S rRNA primer (in bold).
**Table S2:** Thermodynamic characteristics of designed tagged primers. Thermodynamic properties of all primers have been calculated using the OligoAnalyzertool (Integrated DNA Technologies, USA).
**Table S3:** Summary of in‐line barcodes theoretical and experimental percentages (mean ± standard deviation). This table summarizes the theoretical and experimentally observed percentages (mean ± standard deviation) for selected archaeal and bacterial primers. Columns indicate the conditions with zero, one, and two errors, including indels.
**Table S4:** Mapping between numerical identifiers and barcode combinations.
**Table S5:** Diversity indices across archaeal and bacterial sets.
**Table S6:** Summary of selected 96 pairs of in‐line barcodes for archaeal and bacterial sets.
**Table S7:** Accession numbers for all of the sequencing data used in this study.

**Data S2:** Fully annotated R scripts used for all data processing and statistical analyses described in the manuscript.

**Data S3:** DNA concentration measurements obtained after amplicon purification. The “*all*” sheet reports data for all samples combined, while the “*bacteria*” and “*archaea*” sheets correspond to libraries prepared with the bacterial and archaeal barcode sets, respectively.

**Data S4:** Data frame compiling all performance metrics described in Section 3.4, including PCR yield and diversity indices.

**Data S5:** Barcode list for Archaeal and Bacterial primer sets, respectively. Suggested protocol for the use of in‐line barcodes.

## Data Availability

All data supporting the findings of this study, including raw data and metadata, have been made publicly available (Submission ID: PRJEB102343, secondary accession ERP183741). Processing scripts have also been published on GitHub (Lisajrd/16‐pipeline).
