# Supplementary material for: A Scalable and Cost‐Effective In‐Line Barcoding Strategy for Standardized 16S rRNA Gene Amplicon Sequencing: Performance Evaluation and Bias Assessment
Source: Mol Ecol Resour. 2026 May 11;26:e70138. doi: 10.1111/1755-0998.70138 (PMC13159520; doi:10.1111/1755-0998.70138)
Supplement: Supplementary file 1 — Figure S1: Representative quality profile (Sample Zymo, ligation with PCR cycles) of paired‐end reads obtained from the in‐line barcoding workflow. Figure S2: Rarefaction curves showing observed species richness as a function of sequencing depth for all samples. Figure S3: Assessment of PCR yield and amplification consistency across tagged primer pairs. (A) Distribution of DNA concentrations measured by PicoGreen assays for bacterial and archaeal samples. Violin plots show the density of concentration values (ng μL−1), with white diamonds indicating mean values and black bars representing ±1 SD. (B) Representative agarose gel electrophoresis of amplicons from the Archaea_2 sample, amplified with 32 distinct tagged primer combinations: 4 forward barcodes (Forward_Archaea_1–4) each crossed with the same 8 reverse barcodes (Reverse_Archaea_1–8). Lane mapping: 1–8 = Forward_Archaea_1 × Reverse_Archaea_1–8; 9–16 = Forward_Archaea_2 × Reverse_Archaea_1–8; 17–24 = Forward_Archaea_3 × Reverse_Archaea_1–8; 25–32 = Forward_Archaea_4 × Reverse_Archaea_1–8. All reactions yielded single, sharp bands of the expected size (~400 bp). Gels were run in 0.5× TAE buffer on 1% agarose. NC denotes the negative control (PCR run without DNA template). Molecular weight marker: 1 Kb DNA Ladder RTU (GeneDireX Inc.). Figure S4: Normalized read ratios per barcode pair for bacterial and archaeal samples in the “1‐error indel” mode. Dashed red horizontal lines indicate the theoretically expected proportions (1/136 for bacteria, 1/156 for archaea). To account for the fact that only a subset of the full barcode set was used in each sequencing run, read ratios were corrected using a factor reflecting the proportion of barcode combinations tested. Barcode pairs have been replaced by numerical identifiers; a correspondence table is provided in Table S4. Figure S5: Read retention across processing steps. Dot plot showing the number of reads retained per library at each step of the processing pipeline. [file MEN-26-e70138-s003.docx]

A scalable and cost-effective in-line barcoding strategy for standardized 16S rRNA gene amplicon sequencing: performance evaluation and bias assessment

**Authors:** Lisa Jourdain^1^*, Pierre Rossi^2^, Aline Charpagne^3^, Emmanuelle Chevalier^3^, Viviane Praz^3^, Julien Marquis^3^, Johann Webber^3^, Wenyu Gu^1^*

**List of supplementary materials:**

**Figures:**

**Supplementary Materials, Figure 1. Representative quality profile (Sample Zymo, ligation with PCR cycles) of paired-end reads obtained from the in-line barcoding workflow.**

**Supplementary Materials, Figure 2.** **Rarefaction curves showing observed species richness as a function of sequencing depth for all samples.**

**Supplementary Materials, Figure 3.** **Assessment of PCR yield and amplification consistency across tagged primer pairs.** (A) Distribution of DNA concentrations measured by PicoGreen assays for bacterial and archaeal samples. Violin plots show the density of concentration values (ng.µL^-1^), with white diamonds indicating mean values and black bars representing ±1 SD. (B) Representative agarose gel electrophoresis of amplicons from the *Archaea_2* sample, amplified with 32 distinct tagged primer combinations: 4 forward barcodes (Forward_Archaea_1-4) each crossed with the same 8 reverse barcodes (Reverse_Archaea_1-8). Lane mapping: 1-8 = Forward_Archaea_1 × Reverse_Archaea_1-8; 9-16 = Forward_Archaea_2 × Reverse_Archaea_1-8; 17-24 = Forward_Archaea_3 × Reverse_Archaea_1-8; 25-32 = Forward_Archaea_4 × Reverse_Archaea_1-8. All reactions yielded single, sharp bands of the expected size (~400 bp). Gels were run in 0.5× TAE buffer on 1 % agarose. NC denotes the negative control (PCR run without DNA template). Molecular weight marker: 1 Kb DNA Ladder RTU (GeneDireX, Inc.).

**Supplementary Materials, Figure 4. Normalized read ratios per barcode pair for bacterial and archaeal samples in the “1-error indel” mode.** Dashed red horizontal lines indicate the theoretically expected proportions (1/136 for bacteria, 1/156 for archaea). To account for the fact that only a subset of the full barcode set was used in each sequencing run, read ratios were corrected using a factor reflecting the proportion of barcode combinations tested. Barcode pairs have been replaced by numerical identifiers; a correspondence table is provided in **Supplementary Table 4**.

**Supplementary Materials, Figure 5.** **Read retention across processing steps.** Dot plot showing the number of reads retained per library at each step of the processing pipeline.

**Supplementary Materials, Figure 6.** **Relative abundance barplots comparing microbial community composition across barcode combinations in all samples.**  Barcode pairs have been replaced by numerical identifiers; a correspondence table is provided in **Supplementary Table 4.**

**Supplementary Materials, Figure 7. Hierarchical metaclustering of bacterial barcode pairs based on performance similarity across samples.** Dendrogram showing hierarchical clustering (average linkage) of co-clustering frequencies among 136 bacterial barcode pairs. For each sample, performance metrics (diversity metrics and Bray-Curtis distances to a reference barcode pair) were reduced using PCA, and barcode pairs were clustered using k-means (k = 2) in the resulting principal component space. Co-clustering frequencies represent the proportion of samples in which each pair is grouped and were aggregated into a domain-specific matrix. This matrix was transformed into a dissimilarity matrix (1 – co-clustering frequency) and clustered using *hclust* in R.

**Supplementary Materials, Figure 8. Hierarchical metaclustering of archaeal barcode pairs based on performance similarity across samples.** Dendrogram showing hierarchical clustering (average linkage) of co-clustering frequencies among 136 bacterial barcode pairs. For each sample, performance metrics (diversity metrics and Bray-Curtis distances to a reference barcode pair) were reduced using PCA, and barcode pairs were clustered using k-means (k = 2) in the resulting principal component space. Co-clustering frequencies represent the proportion of samples in which each pair is grouped and were aggregated into a domain-specific matrix. This matrix was transformed into a dissimilarity matrix (1 – co-clustering frequency) and clustered using *hclust* in R.

**Supplementary Materials, Figure 9. Comparison of species-level relative abundance across barcode combinations in individual and pooled library preparations.**

**Supplementary Materials, Figure 10. Archaeal profiles with selected tagged barcode pairs.** (**A)** Bray-Curtis distance to a randomly selected barcode pair across the selected barcode combinations for all bacterial samples. (**B)** Taxonomic profile of sample Archaea 3 across the selected barcode pairs using the PCR-free ligation protocol.

**Tables:**

**Supplementary Materials Table 1. Sequences of custom-designed tagged primers for 16S rRNA gene sequencing.** Each primer includes a unique barcode tag, the leader sequence (underlined), and a 16S rRNA primer (in **bold**).

**Supplementary Materials Table 2. Thermodynamic characteristics of designed tagged primers.** Thermodynamic properties of all primers have been calculated using the OligoAnalyzer™tool (Integrated DNA Technologies, USA).

**Supplementary Materials, Table 3. Summary of in-line barcodes theoretical and experimental percentages (mean ± standard deviation).** This table summarizes the theoretical and experimentally observed percentages (mean ± standard deviation) for selected archaeal and bacterial primers. Columns indicate the conditions with zero, one, and two errors, including indels.

**Supplementary** **Materials, Table 4**. **Mapping between numerical identifiers and barcode combinations.**

**Supplementary Materials, Table 5. Diversity indices across archaeal and bacterial sets.** *This table is not presented in this document due to its size but is available as an Excel file.*

**Supplementary Materials, Table 6. Summary of selected 96 pairs of in-line barcodes for archaeal and bacterial sets.**

**Supplementary Materials, Table 7 Accession numbers for all of the sequencing data used in this study.**

### ****Files:****

- **Cleaned_Annotated_Script.Rmd**: Fully annotated R scripts used for all data processing and statistical analyses described in the manuscript.
- **Picogreen.xlsx**: DNA concentration measurements obtained after amplicon purification. The “all” sheet reports data for all samples combined, while the “bacteria” and “archaea” sheets correspond to libraries prepared with the bacterial and archaeal barcode sets, respectively.
- **Metrics.xlsx**: Data frame compiling all performance metrics described in **Section 4.4**, including PCR yield and diversity indices.
- **Arch_F.fasta, Arch_R.fasta, Bact_F.fasta, Bact_R.fasta**: Barcode list for Archaeal and Bacterial primer sets, respectively.
- **Short_protocol.pdf**: Suggested protocol for the use of in-line barcodes.

**Supplementary Figures**

**
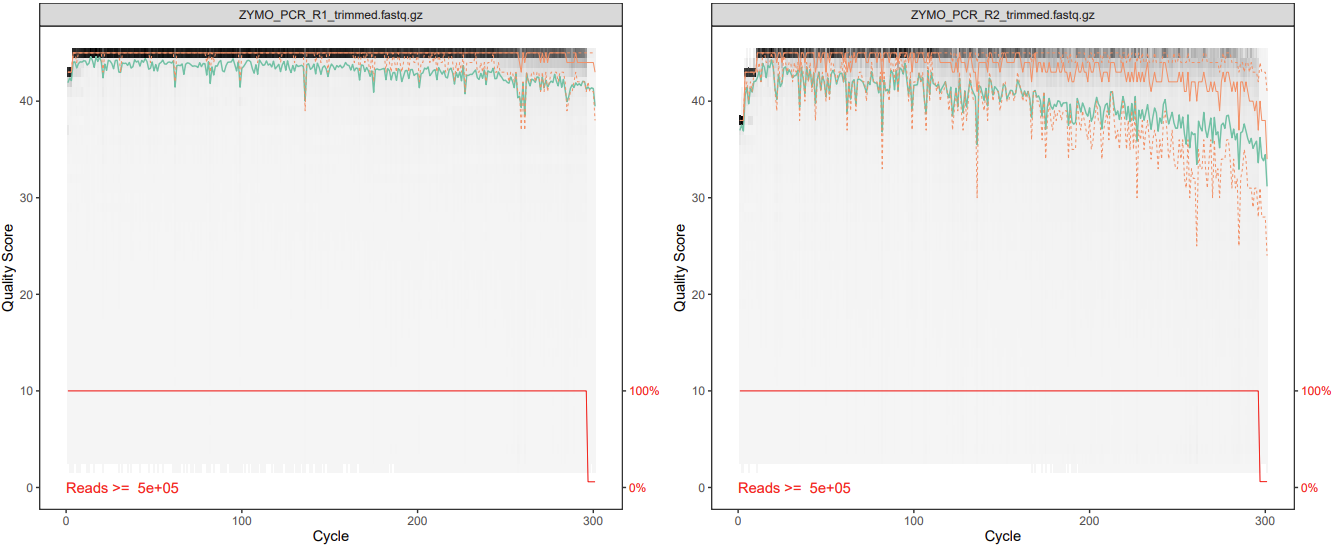
**

**Supplementary Materials, Figure 1.** **Representative quality profile (Sample Zymo, ligation with PCR cycles) of paired-end reads obtained from the in-line barcoding workflow.**

**
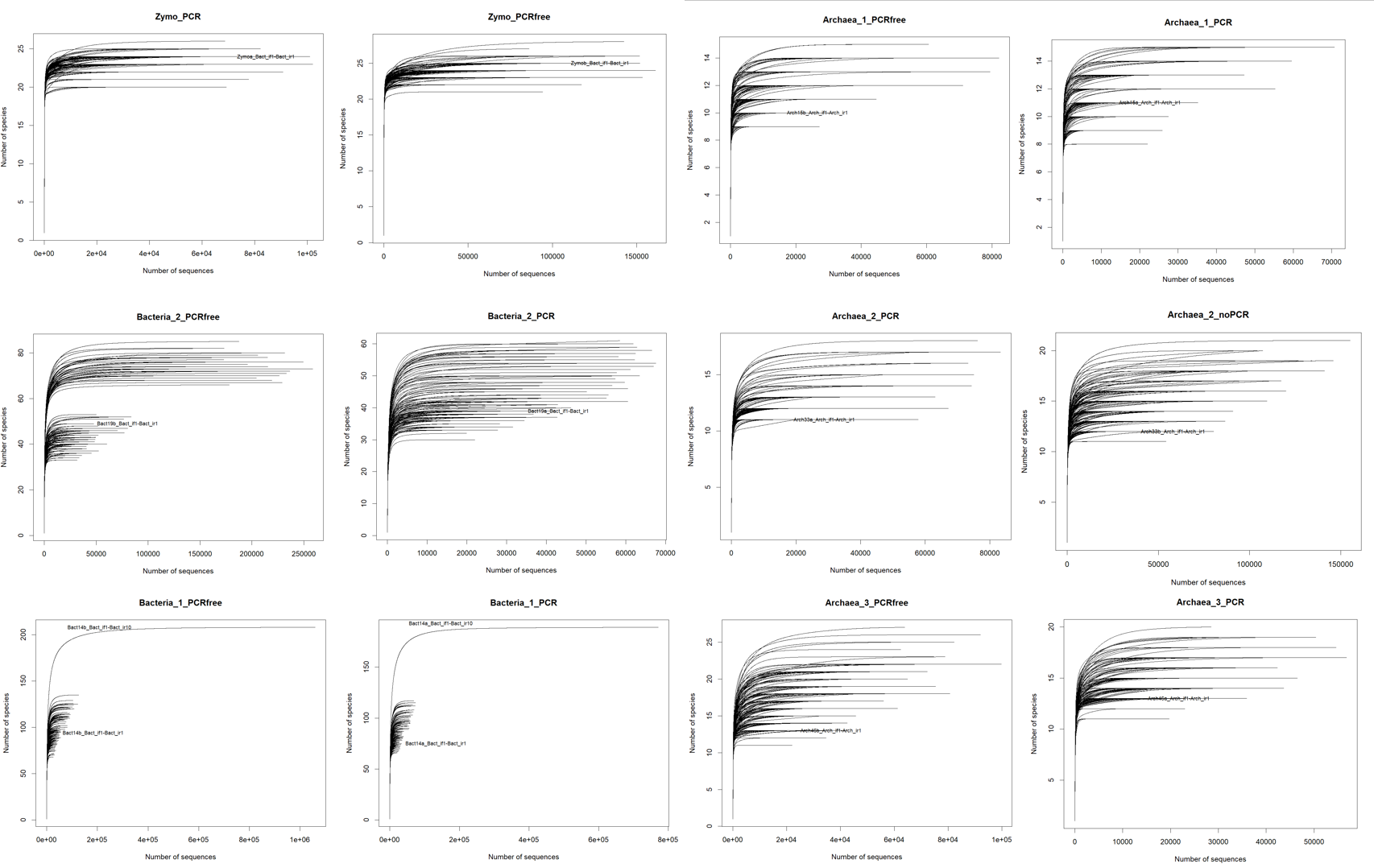
**

**Supplementary Materials, Figure 2.** **Rarefaction curves showing observed species richness as a function of sequencing depth for all samples.**


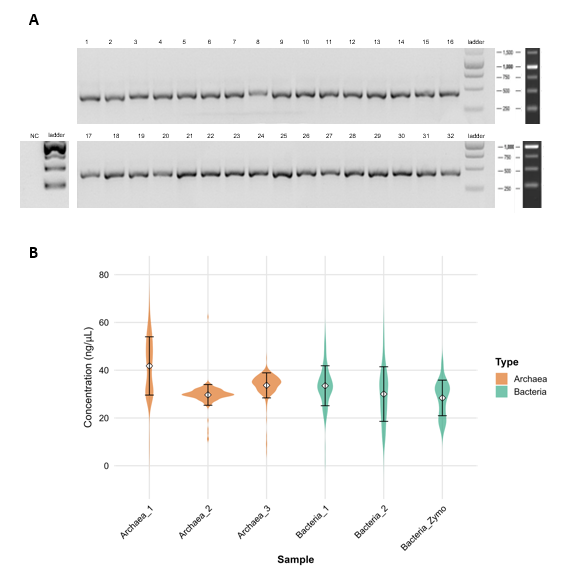


**Supplementary Materials, Figure 3.** **Assessment of PCR yield and amplification consistency across tagged primer pairs.**  **(A)** **Representative agarose gel electrophoresis of amplicons from the Archaea_2 sample, amplified with** **32 distinct tagged primer combinations**: 4 forward barcodes (Forward_Archaea_1-4) each crossed with the same 8 reverse barcodes (Reverse_Archaea_1-8). Lane mapping: 1-8 = Forward_Archaea_1 × Reverse_Archaea_1-8; 9-16 = Forward_Archaea_2 × Reverse_Archaea_1-8; 17-24 = Forward_Archaea_3 × Reverse_Archaea_1-8; 25-32 = Forward_Archaea_4 × Reverse_Archaea_1-8. All reactions yielded single, sharp bands of the expected size (~400 bp). Gels were run in 0.5× TAE buffer on 1 % agarose. NC denotes the negative control (PCR run without DNA template). Molecular weight marker: 1 Kb DNA Ladder RTU (GeneDireX, Inc.).  **(B)** **Distribution of DNA concentrations measured by PicoGreen assays for bacterial and archaeal samples**. Violin plots show the density of concentration values (ng.µL⁻¹), with white diamonds indicating mean values and black bars representing ±1 SD.


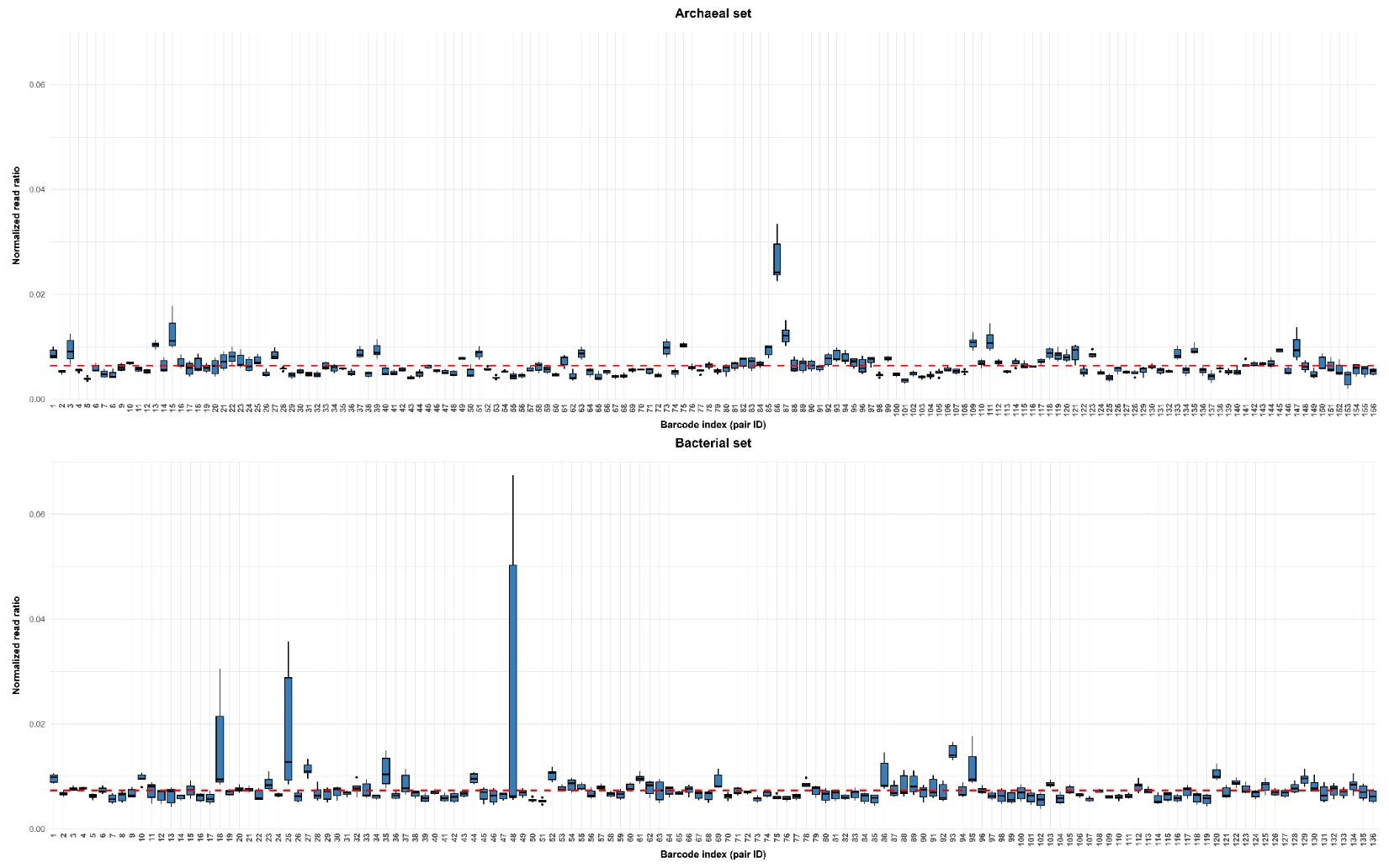


**Supplementary Materials, Figure 4. Normalized read ratios per barcode pair for bacterial and archaeal samples in the “1-error indel” mode.** Dashed red horizontal lines indicate the theoretically expected proportions (1/136 for bacteria, 1/156 for archaea). To account for the fact that only a subset of the full barcode set was used in each sequencing run, read ratios were corrected using a factor reflecting the proportion of barcode combinations tested. Barcode pairs have been replaced by numerical identifiers; a correspondence table is provided in **Supplementary Table 4**.


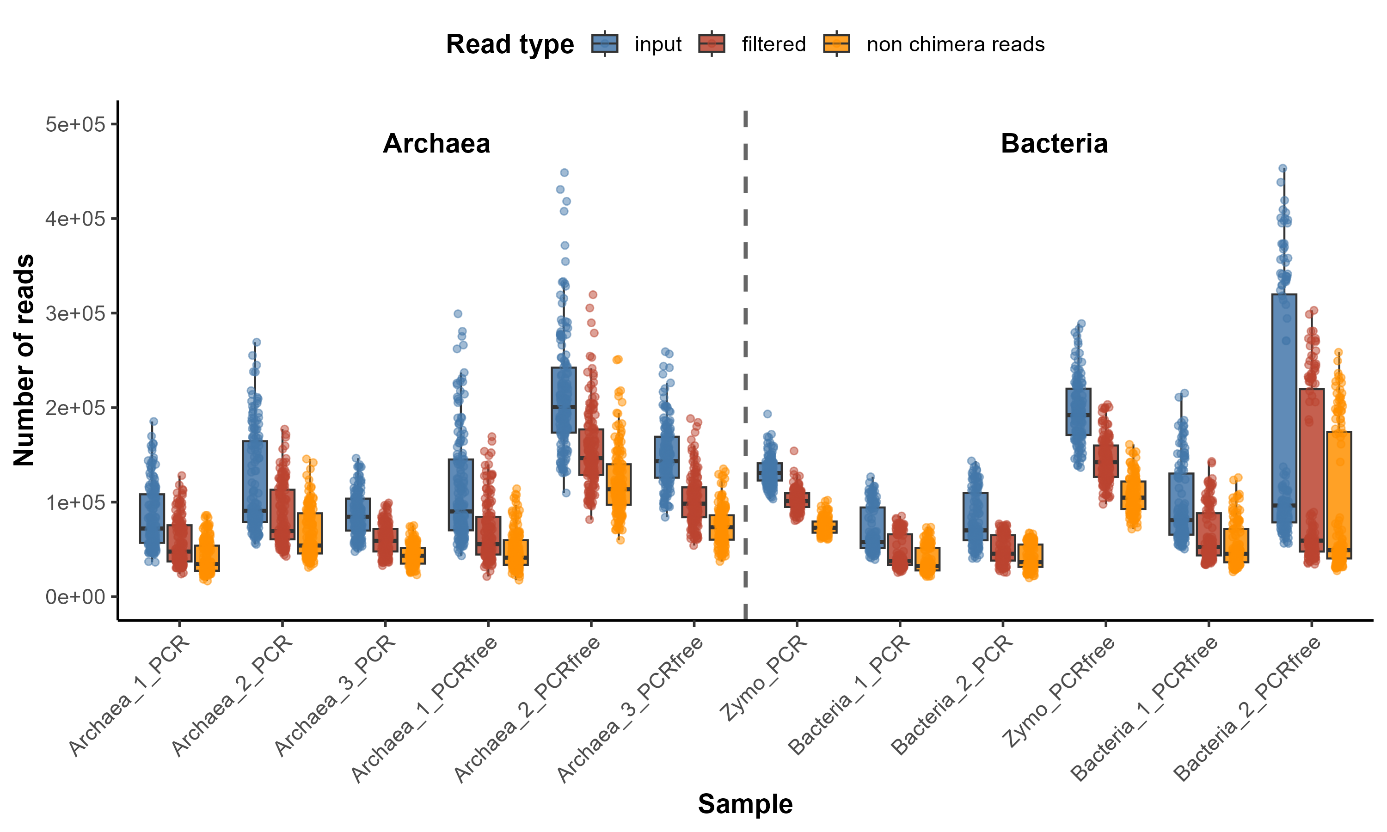


**Supplementary Materials, Figure 5.** **Read retention across processing steps.** Dot plot showing the number of reads retained per library at each step of the processing pipeline.


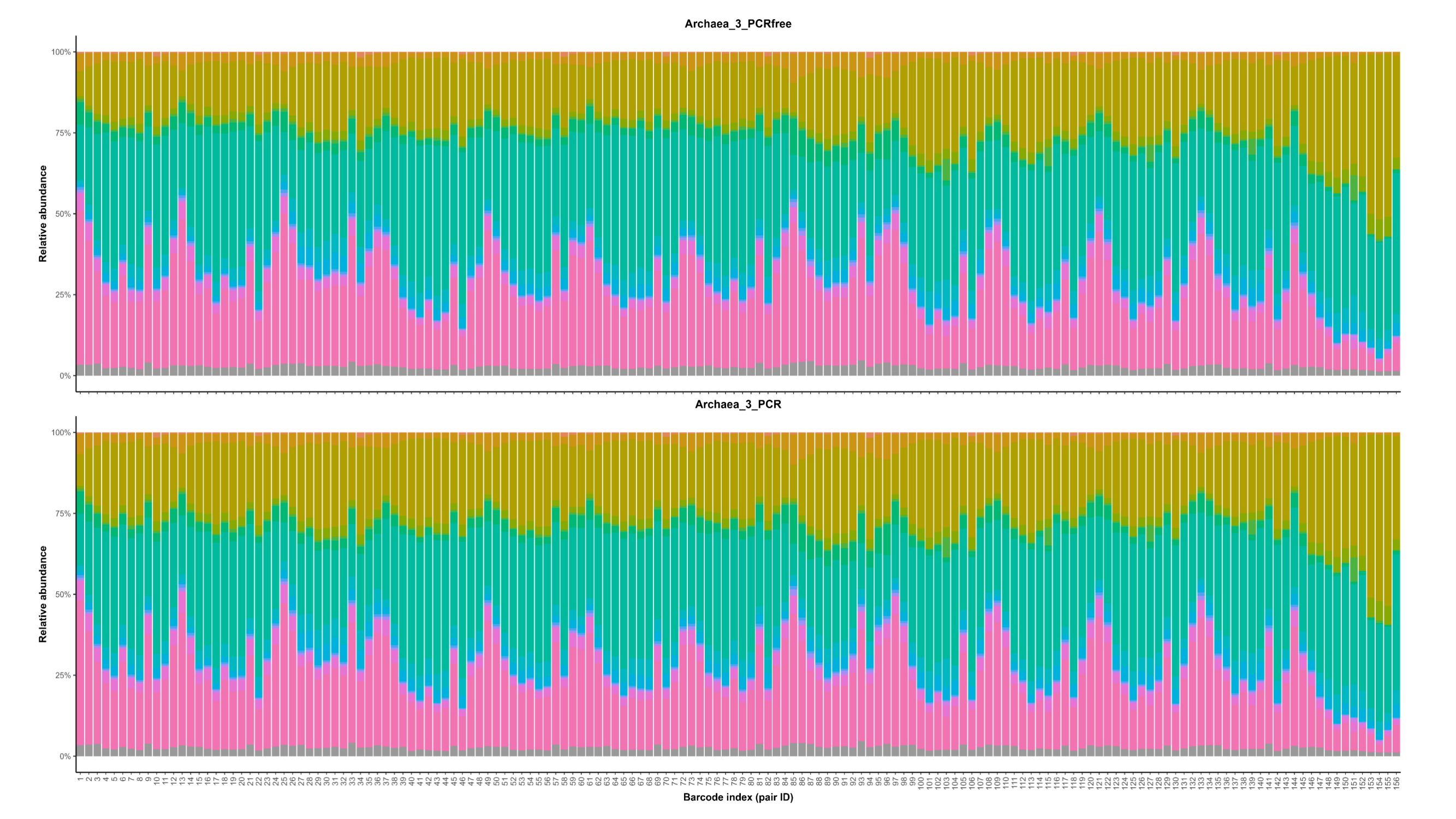

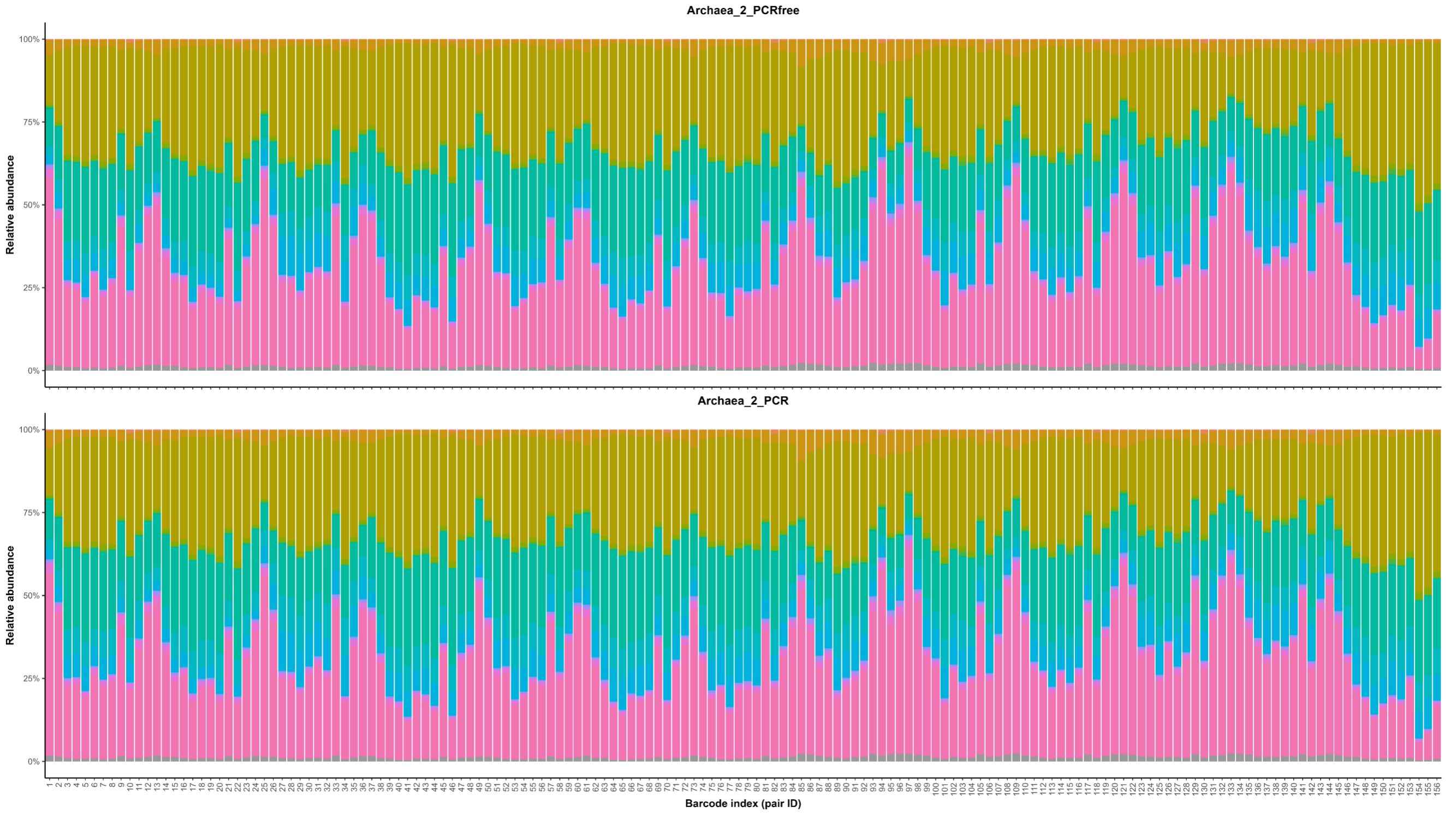

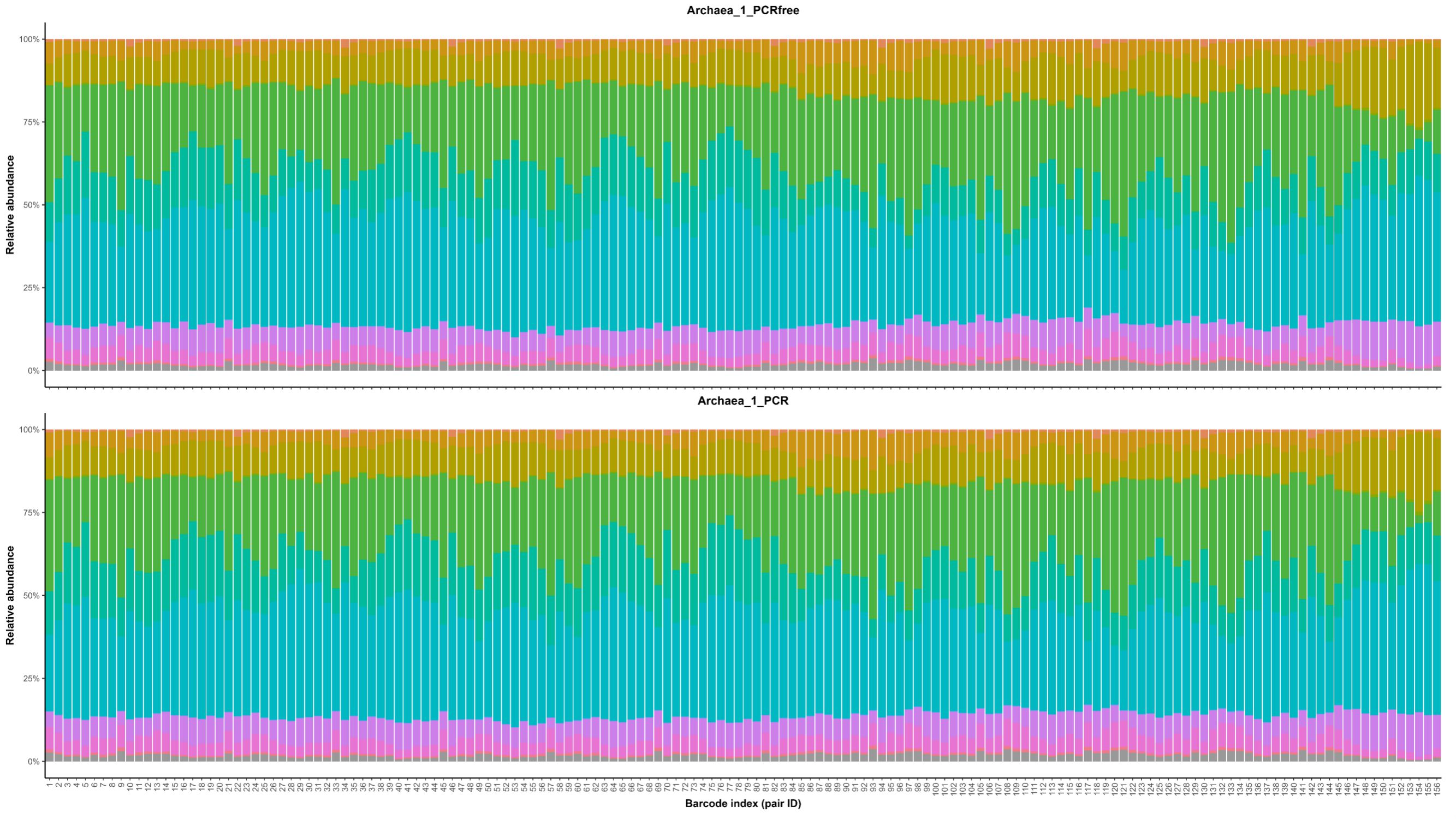

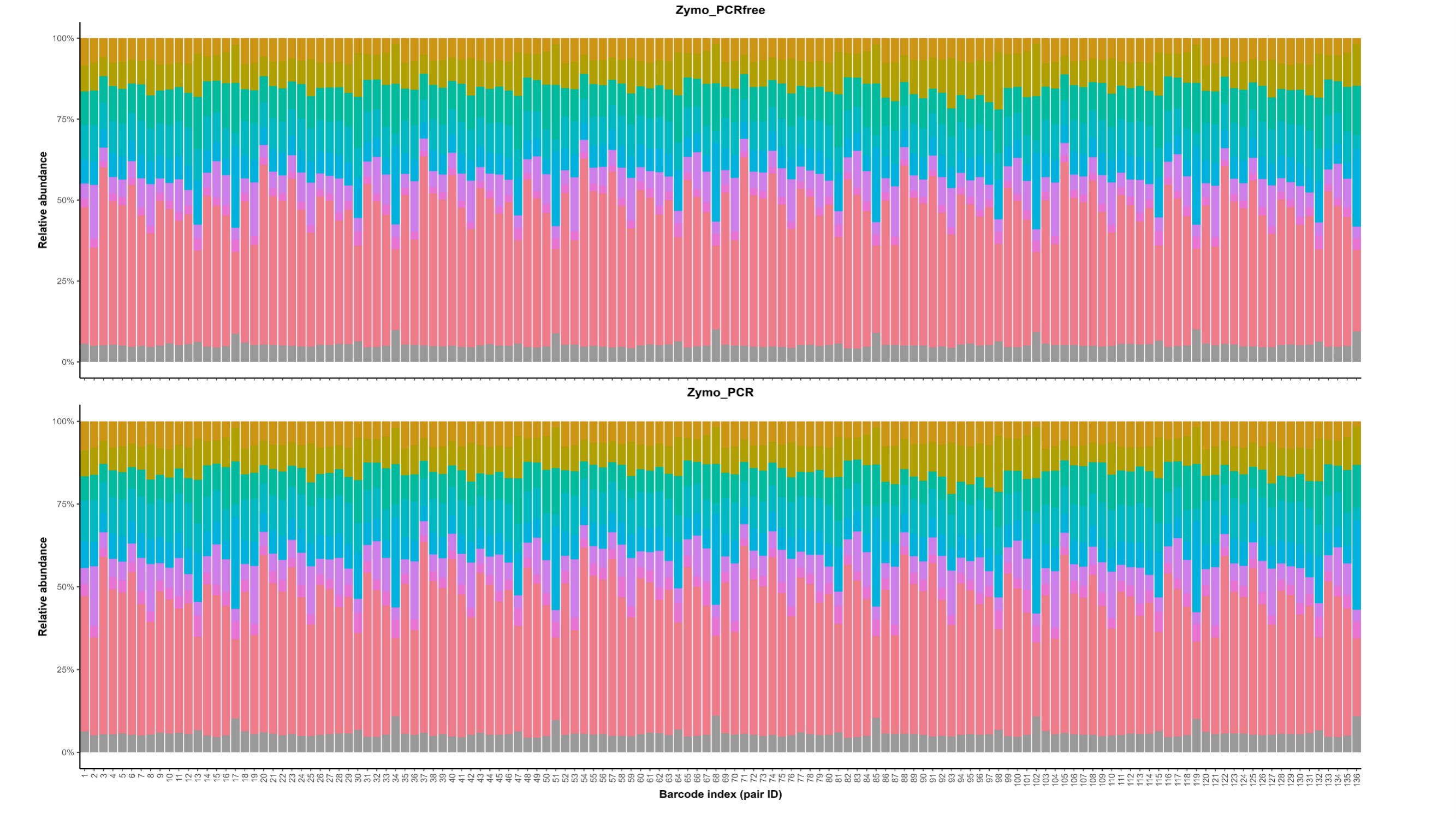

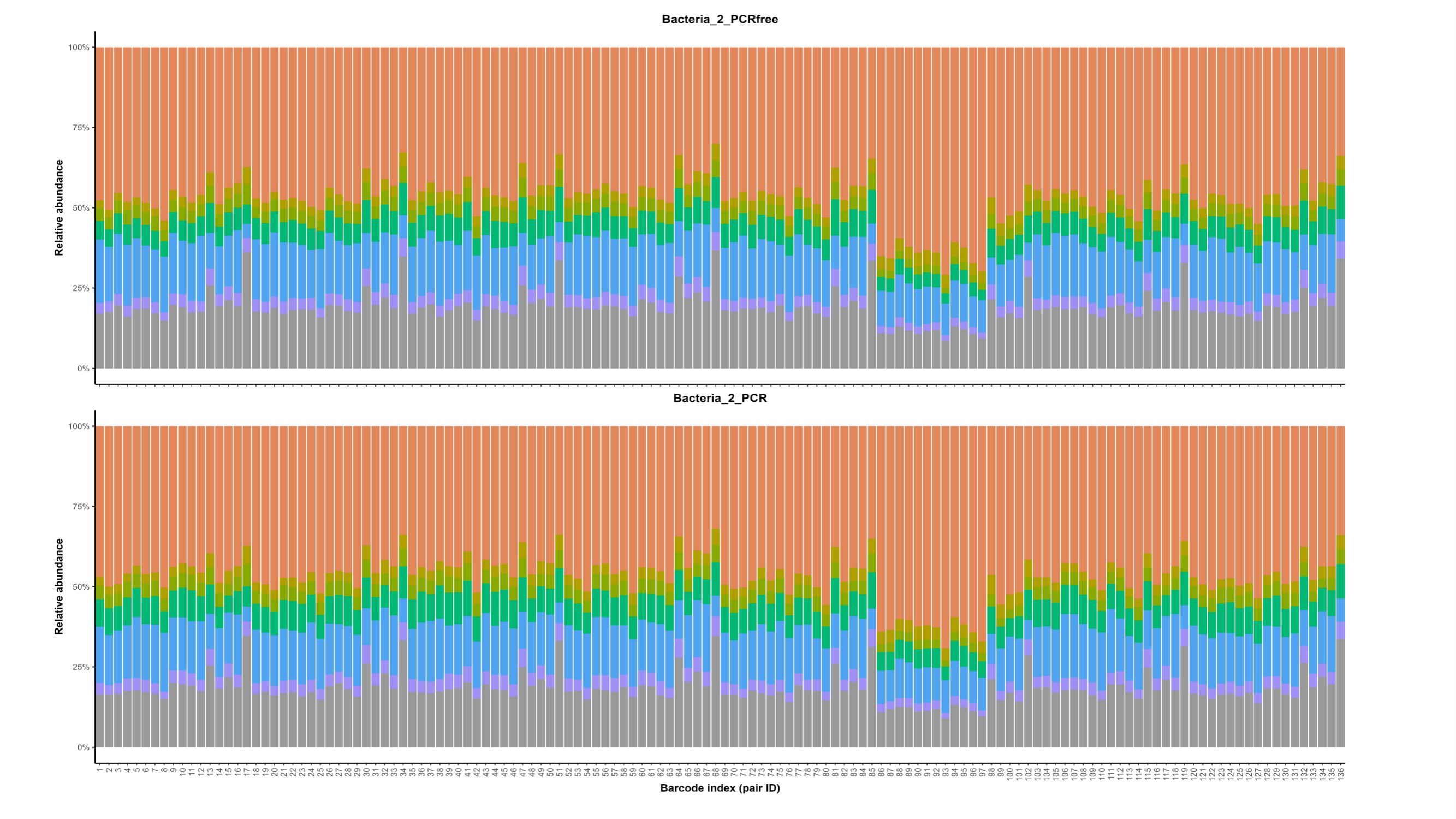

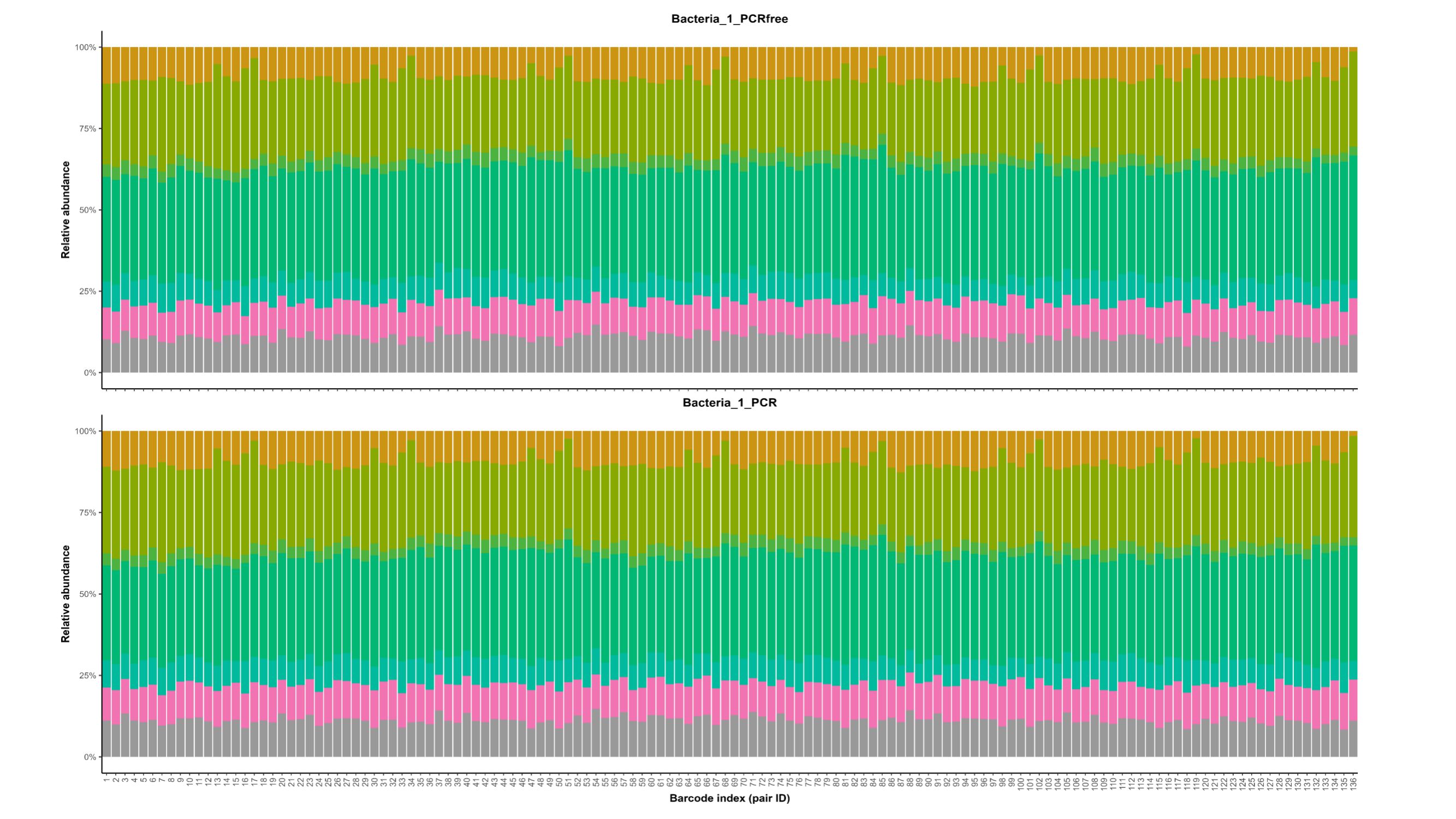


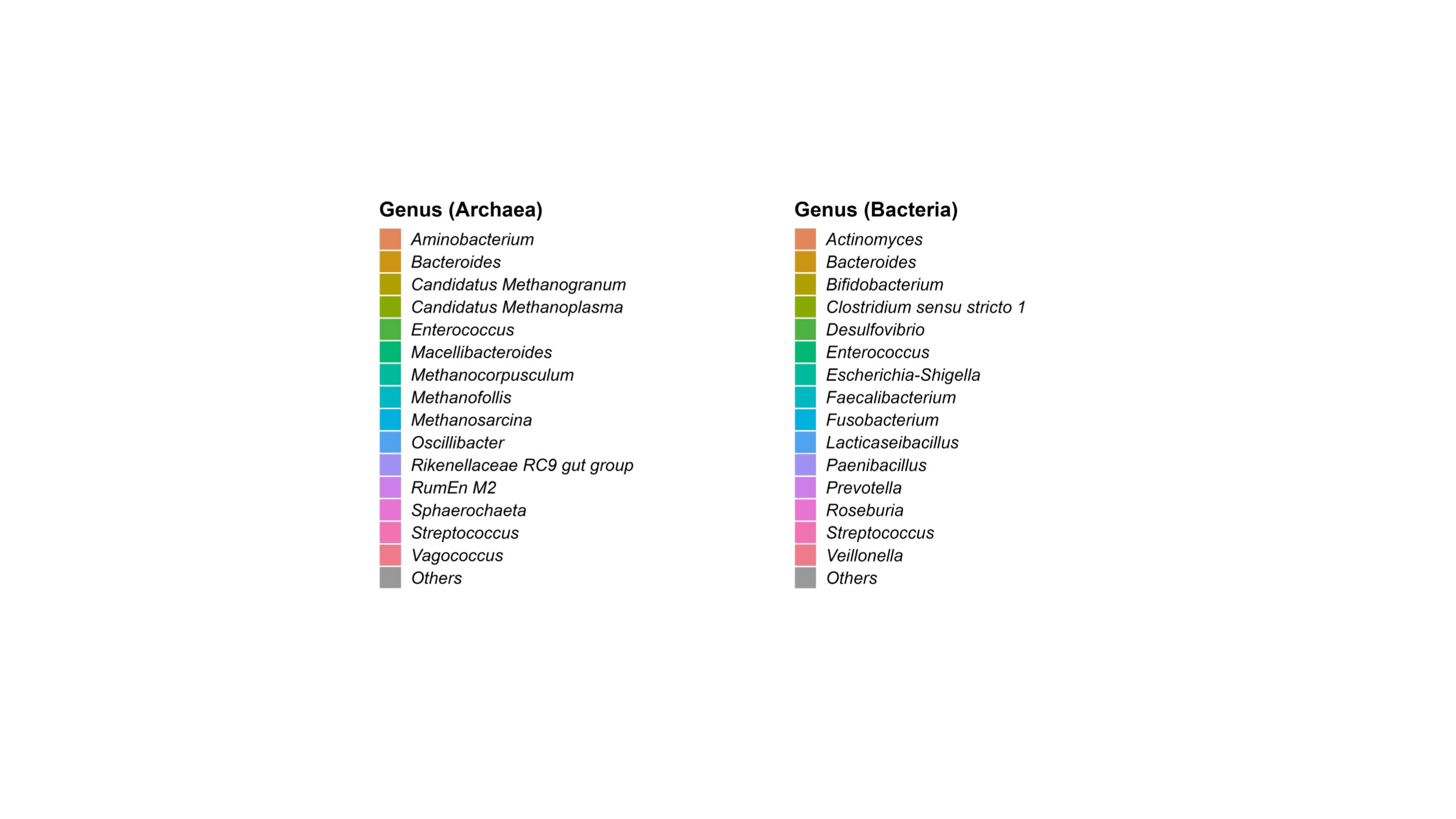


**Supplementary Materials, Figure 6.** **Relative abundance barplots comparing microbial community composition across barcode combinations in all samples.**


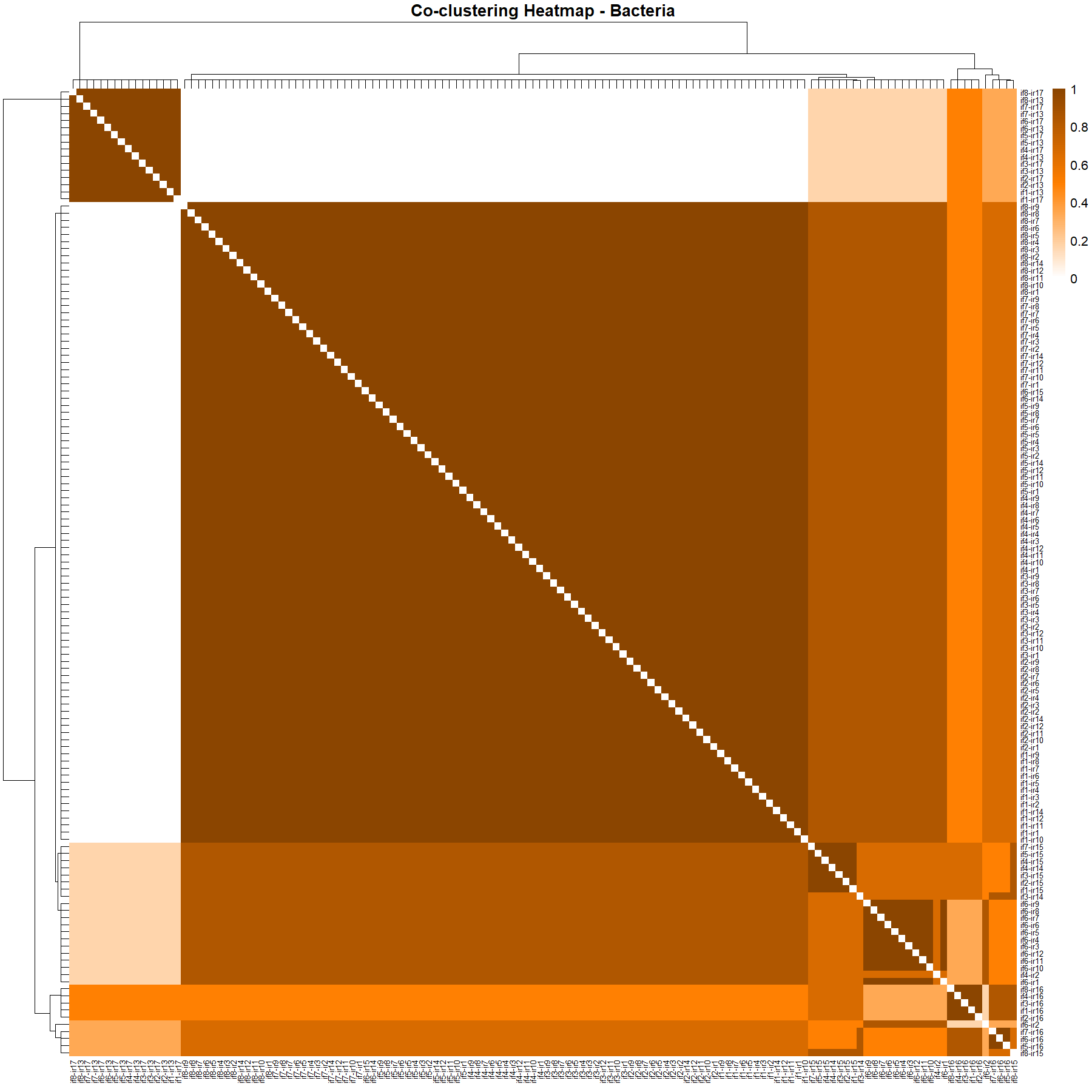


**Supplementary Materials, Figure 7. Hierarchical metaclustering of bacterial barcode pairs based on performance similarity across samples.** Dendrogram showing hierarchical clustering (average linkage) of co-clustering frequencies among 136 bacterial barcode pairs. For each sample, performance metrics (diversity metrics and Bray-Curtis distances to a reference barcode pair) were reduced using PCA, and barcode pairs were clustered using k-means (k = 2) in the resulting principal component space. Co-clustering frequencies represent the proportion of samples in which each pair is grouped and were aggregated into a domain-specific matrix. This matrix was transformed into a dissimilarity matrix (1 – co-clustering frequency) and clustered using *hclust* in R.


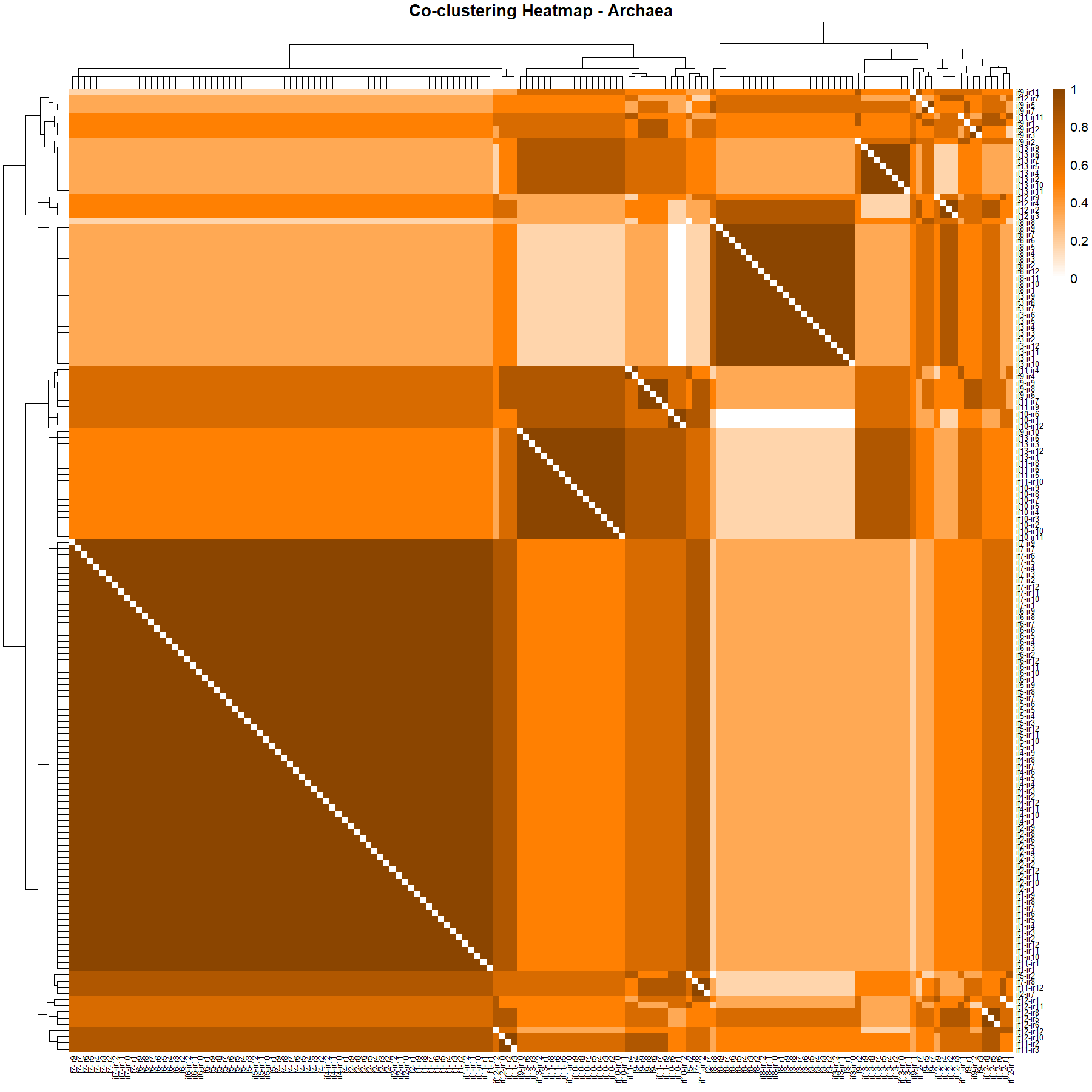


**Supplementary Materials, Figure 8. Hierarchical metaclustering of archaeal barcode pairs based on performance similarity across samples.** Dendrogram showing hierarchical clustering (average linkage) of co-clustering frequencies among 136 bacterial barcode pairs. For each sample, performance metrics (diversity metrics and Bray-Curtis distances to a reference barcode pair) were reduced using PCA, and barcode pairs were clustered using k-means (k = 2) in the resulting principal component space. Co-clustering frequencies represent the proportion of samples in which each pair is grouped and were aggregated into a domain-specific matrix. This matrix was transformed into a dissimilarity matrix (1 – co-clustering frequency) and clustered using *hclust* in R.


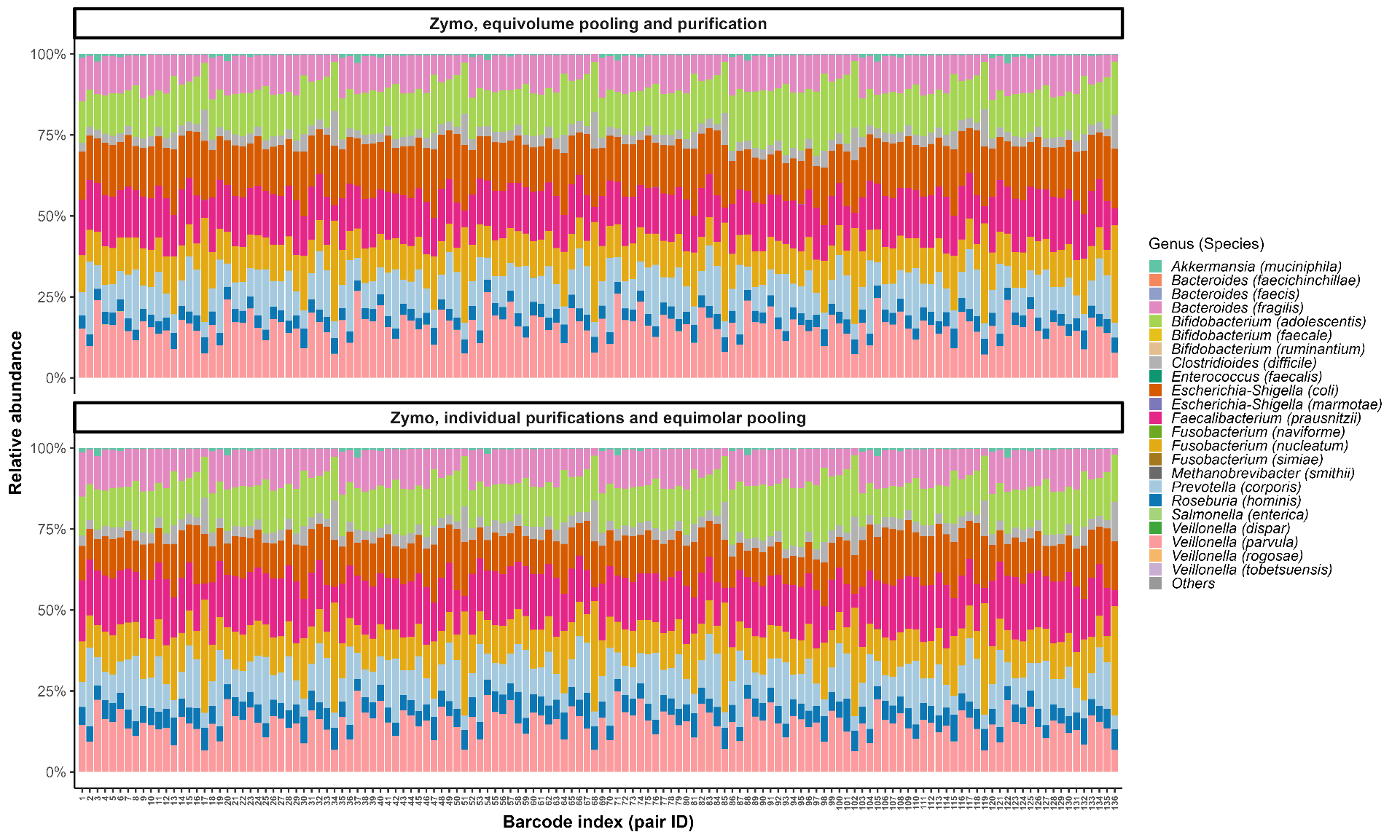


**Supplementary Materials, Figure 9. Comparison of species-level relative abundance across all barcode combinations in individual and pooled library preparations.** Barcode pairs have been replaced by numerical identifiers; a correspondence table is provided in **Supplementary Table 4**.


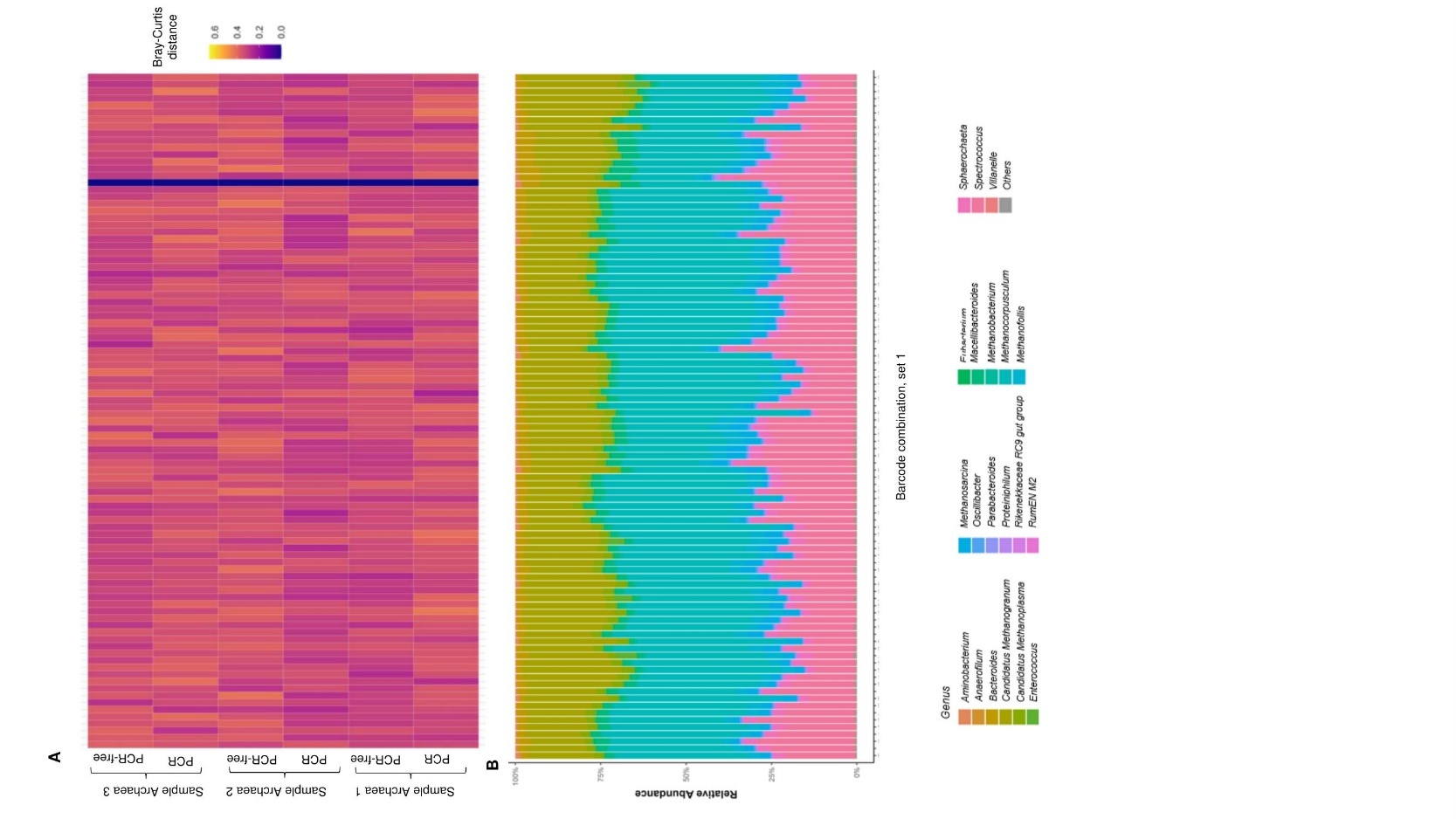


**Supplementary Materials, Figure 10. Archaeal profiles with selected tagged barcode pairs.** (**A)** Bray-Curtis distance to a randomly selected barcode pair across the selected barcode combinations for all bacterial samples. (**B)** Taxonomic profile of sample Archaea 3 across the selected barcode pairs using the PCR-free ligation protocol.

**Supplementary Tables**

**Supplementary Materials Table 1. Sequences of custom-designed tagged primers for 16S rRNA gene sequencing.** Each primer includes a unique barcode tag, the leader sequence (underlined), and a 16S rRNA primer (in **bold**).

| Domain | Orientation | Names | Sequence (5'->3' ) |
| --- | --- | --- | --- |
| Archaea | Forward | Forward_Archaea_1 | GCATCCTCTCTAT**CAGCCGCCGCGGTAA** |
|  |  | Forward_Archaea_2 | GCATCTATCCTCT**CAGCCGCCGCGGTAA** |
|  |  | Forward_Archaea_3 | GCATCGTAAGGAG**CAGCCGCCGCGGTAA** |
|  |  | Forward_Archaea_4 | GCATCACTGCATA**CAGCCGCCGCGGTAA** |
|  |  | Forward_Archaea_5 | GCATCAAGGAGTA**CAGCCGCCGCGGTAA** |
|  |  | Forward_Archaea_6 | GCATCCTAAGCCT**CAGCCGCCGCGGTAA** |
|  |  | Forward_Archaea_7 | GCATCCGTCTAAT**CAGCCGCCGCGGTAA** |
|  |  | Forward_Archaea_8 | GCATCTCTCTCCG**CAGCCGCCGCGGTAA** |
|  |  | Forward_Archaea_9 | GCATCTCGACTAG**CAGCCGCCGCGGTAA** |
|  |  | Forward_Archaea_10 | GCATCTTCTAGCT**CAGCCGCCGCGGTAA** |
|  |  | Forward_Archaea_11 | GCATCCCTAGAGT**CAGCCGCCGCGGTAA** |
|  |  | Forward_Archaea_12 | GCATCGCGTAAGA**CAGCCGCCGCGGTAA** |
|  |  | Forward_Archaea_13 | GCATCCTATTAAG**CAGCCGCCGCGGTAA** |
|  | Reverse | Reverse_Archaea_1 | GCATCTCGCCTTA**GTGCTCCCCCGCCAATTCCT** |
|  |  | Reverse_Archaea_2 | GCATCCTAGTACG**GTGCTCCCCCGCCAATTCCT** |
|  |  | Reverse_Archaea_3 | GCATCTTCTGCCT**GTGCTCCCCCGCCAATTCCT** |
|  |  | Reverse_Archaea_4 | GCATCGCTCAGGA**GTGCTCCCCCGCCAATTCCT** |
|  |  | Reverse_Archaea_5 | GCATCAGGAGTCC**GTGCTCCCCCGCCAATTCCT** |
|  |  | Reverse_Archaea_6 | GCATCCATGCCTA**GTGCTCCCCCGCCAATTCCT** |
|  |  | Reverse_Archaea_7 | GCATCGTAGAGAG**GTGCTCCCCCGCCAATTCCT** |
|  |  | Reverse_Archaea_8 | GCATCCAGCCTCG**GTGCTCCCCCGCCAATTCCT** |
|  |  | Reverse_Archaea_9 | GCATCTGCCTCTT**GTGCTCCCCCGCCAATTCCT** |
|  |  | Reverse_Archaea_10 | GCATCTCCTCTAC**GTGCTCCCCCGCCAATTCCT** |
|  |  | Reverse_Archaea_11 | GCATCTCATGAGC**GTGCTCCCCCGCCAATTCCT** |
|  |  | Reverse_Archaea_12 | GCATCCCTGAGAT**GTGCTCCCCCGCCAATTCCT** |
| Bacteria | Forward | Forward_Bacteria_1 | GCATCCTCTCTAT**ACTCCTACGGGAGGCAGCAG** |
|  |  | Forward_Bacteria_2 | GCATCTATCCTCT**ACTCCTACGGGAGGCAGCAG** |
|  |  | Forward_Bacteria_3 | GCATCGTAAGGAG**ACTCCTACGGGAGGCAGCAG** |
|  |  | Forward_Bacteria_4 | GCATCACTGCATA**ACTCCTACGGGAGGCAGCAG** |
|  |  | Forward_Bacteria_5 | GCATCAAGGAGTA**ACTCCTACGGGAGGCAGCAG** |
|  |  | Forward_Bacteria_6 | GCATCCTAAGCCT**ACTCCTACGGGAGGCAGCAG** |
|  |  | Forward_Bacteria_7 | GCATCCGTCTAAT**ACTCCTACGGGAGGCAGCAG** |
|  |  | Forward_Bacteria_8 | GCATCTCTCTCCG**ACTCCTACGGGAGGCAGCAG** |
|  | Reverse | Reverse_Bacteria_1 | GCATCTCGCCTTA**GACTACHVGGGTWTCTAAT** |
|  |  | Reverse_Bacteria_2 | GCATCCTAGTACG**GACTACHVGGGTWTCTAAT** |
|  |  | Reverse_Bacteria_3 | GCATCTTCTGCCT**GACTACHVGGGTWTCTAAT** |
|  |  | Reverse_Bacteria_4 | GCATCGCTCAGGA**GACTACHVGGGTWTCTAAT** |
|  |  | Reverse_Bacteria_5 | GCATCAGGAGTCC**GACTACHVGGGTWTCTAAT** |
|  |  | Reverse_Bacteria_6 | GCATCCATGCCTA**GACTACHVGGGTWTCTAAT** |
|  |  | Reverse_Bacteria_7 | GCATCGTAGAGAG**GACTACHVGGGTWTCTAAT** |
|  |  | Reverse_Bacteria_8 | GCATCCAGCCTCG**GACTACHVGGGTWTCTAAT** |
|  |  | Reverse_Bacteria_9 | GCATCTGCCTCTT**GACTACHVGGGTWTCTAAT** |
|  |  | Reverse_Bacteria_10 | GCATCTCCTCTAC**GACTACHVGGGTWTCTAAT** |
|  |  | Reverse_Bacteria_11 | GCATCTCATGAGC**GACTACHVGGGTWTCTAAT** |
|  |  | Reverse_Bacteria_12 | GCATCCCTGAGAT**GACTACHVGGGTWTCTAAT** |
|  |  | Reverse_Bacteria_13 | GCATCTAGCGAGT**GACTACHVGGGTWTCTAAT** |
|  |  | Reverse_Bacteria_14 | GCATCGTAGCTCC**GACTACHVGGGTWTCTAAT** |
|  |  | Reverse_Bacteria_15 | GCATCTACTACGC**GACTACHVGGGTWTCTAAT** |
|  |  | Reverse_Bacteria_16 | GCATCAGGCTCCG**GACTACHVGGGTWTCTAAT** |
|  |  | Reverse_Bacteria_17 | GCATCGCAGCGTA**GACTACHVGGGTWTCTAAT** |

**Supplementary Materials Table 2. Thermodynamic characteristics of designed tagged primers.** Thermodynamic properties of all primers have been calculated using the OligoAnalyzer™tool (Integrated DNA Technologies, USA).

| Name | Sequence | Length | % GC | ΔG | Tm (°C) |
| --- | --- | --- | --- | --- | --- |
| Reverse_Bacteria_1 | GCA TCT CGC CTT AGA CTA CHV GGG TWT CTA AT | 32 | 46.9 | -2.97 | 43.2 |
| Reverse_Bacteria_2 | GCA TCC TAG TAC GGA CTA CHV GGG TWT CTA AT | 32 | 46.9 | -2.5 | 47.7 |
| Reverse_Bacteria_3 | GCA TCT TCT GCC TGA CTA CHV GGG TWT CTA AT | 32 | 46.9 | -2.38 | 39.8 |
| Reverse_Bacteria_4 | GCA TCG CTC AGG AGA CTA CHV GGG TWT CTA AT | 32 | 50 | -1.8 | 49.1 |
| Reverse_Bacteria_5 | GCA TCA GGA GTC CGA CTA CHV GGG TWT CTA AT | 32 | 50 | -1.4 | 40.5 |
| Reverse_Bacteria_6 | GCA TCC ATG CCT AGA CTA CHV GGG TWT CTA AT | 32 | 46.9 | -3.94 | 47.7 |
| Reverse_Bacteria_7 | GCA TCG TAG AGA GGA CTA CHV GGG TWT CTA AT | 32 | 46.9 | -2.72 | 58.7 |
| Reverse_Bacteria_8 | GCA TCC AGC CTC GGA CTA CHV GGG TWT CTA AT | 32 | 53.1 | -1.41 | 35.6 |
| Reverse_Bacteria_9 | GCA TCT GCC TCT TGA CTA CHV GGG TWT CTA AT | 32 | 46.9 | -1.42 | 34.7 |
| Reverse_Bacteria_10 | GCA TCT CCT CTA CGA CTA CHV GGG TWT CTA AT | 32 | 46.9 | -0.61 | 32.2 |
| Reverse_Bacteria_11 | GCA TCT CAT GAG CGA CTA CHV GGG TWT CTA AT | 32 | 46.9 | -1.64 | 35.9 |
| Reverse_Bacteria_12 | GCA TCC CTG AGA TGA CTA CHV GGG TWT CTA AT | 32 | 46.9 | -3.32 | 57.8 |
| Reverse_Bacteria_13 | GCA TCT AGC GAG TGA CTA CHV GGG TWT CTA AT | 32 | 46.9 | -1.59 | 41 |
| Reverse_Bacteria_14 | GCA TCG TAG CTC CGA CTA CHV GGG TWT CTA AT | 32 | 50 | -1.22 | 37.6 |
| Reverse_Bacteria_15 | GCA TCT ACT ACG CGA CTA CHV GGG TWT CTA AT | 32 | 46.9 | -0.61 | 32.2 |
| Reverse_Bacteria_16 | GCA TCA GGC TCC GGA CTA CHV GGG TWT CTA AT | 32 | 53.1 | -1.62 | 36.5 |
| Reverse_Bacteria_17 | GCA TCG CAG CGT AGA CTA CHV GGG TWT CTA AT | 32 | 50 | -2.11 | 47.7 |
| Forward_Bacteria_1 | GCA TCC TCT CTA TAC TCC TAC GGG AGG CAG CAG | 33 | 57,6 | -4 | 58,1 |
| Forward_Bacteria_2 | GCA TCT ATC CTC TAC TCC TAC GGG AGG CAG CAG | 33 | 57,6 | -4 | 58,1 |
| Forward_Bacteria_3 | GCA TCG TAA GGA GAC TCC TAC GGG AGG CAG CAG | 33 | 60,6 | -4,52 | 51,9 |
| Forward_Bacteria_4 | GCA TCA CTG CAT AAC TCC TAC GGG AGG CAG CAG | 33 | 57,6 | -5,91 | 53,8 |
| Forward_Bacteria_5 | GCA TCA AGG AGT AAC TCC TAC GGG AGG CAG CAG | 33 | 57,6 | -4 | 58,1 |
| Forward_Bacteria_6 | GCA TCC TAA GCC TAC TCC TAC GGG AGG CAG CAG | 33 | 60,6 | -4,23 | 63,6 |
| Forward_Bacteria_7 | GCA TCC GTC TAA TAC TCC TAC GGG AGG CAG CAG | 33 | 57,6 | -4 | 58,1 |
| Forward_Bacteria_8 | GCA TCT CTC TCC GAC TCC TAC GGG AGG CAG CAG | 33 | 63,6 | -4 | 58,1 |
| Forward_Archaea_1 | GCA TCC TCT CTA TCA GCC GCC GCG GTA A | 28 | 60.7 | -2.12 | 49.4 |
| Forward_Archaea_2 | GCA TCT ATC CTC TCA GCC GCC GCG GTA A | 28 | 60.7 | -2.12 | 49.4 |
| Forward_Archaea_3 | GCA TCG TAA GGA GCA GCC GCC GCG GTA A | 28 | 64.3 | -2.64 | 44.7 |
| Forward_Archaea_4 | GCA TCA CTG CAT ACA GCC GCC GCG GTA A | 28 | 60.7 | -4.03 | 48.5 |
| Forward_Archaea_5 | GCA TCA AGG AGT ACA GCC GCC GCG GTA A | 28 | 60.7 | -2.12 | 49.4 |
| Forward_Archaea_6 | GCA TCC TAA GCC TCA GCC GCC GCG GTA A | 28 | 64.3 | -2.12 | 49.4 |
| Forward_Archaea_7 | GCA TCC GTC TAA TCA GCC GCC GCG GTA A | 28 | 60.7 | -2.12 | 49.4 |
| Forward_Archaea_8 | GCA TCT CTC TCC GCA GCC GCC GCG GTA A | 28 | 67.9 | -4.39 | 63.1 |
| Forward_Archaea_9 | GCA TCT CGA CTA GCA GCC GCC GCG GTA A | 28 | 64.3 | -2.37 | 41 |
| Forward_Archaea_10 | GCA TCT TCT AGC TCA GCC GCC GCG GTA A | 28 | 60.7 | -2.12 | 49.4 |
| Forward_Archaea_11 | GCA TCC CTA GAG TCA GCC GCC GCG GTA A | 28 | 64.3 | -2.12 | 49.4 |
| Forward_Archaea_12 | GCA TCG CGT AAG ACA GCC GCC GCG GTA A | 28 | 64.3 | -4.51 | 61.5 |
| Forward_Archaea_13 | GCA TCC TAT TAA GCA GCC GCC GCG GTA A | 28 | 57.1 | -2.37 | 41 |
| Reverse_Archaea_1 | GCA TCT CGC CTT AGT GCT CCC CCG CCA ATT CCT | 33 | 60.6 | -0.94 | 35.8 |
| Reverse_Archaea_2 | GCA TCC TAG TAC GGT GCT CCC CCG CCA ATT CCT | 33 | 60.6 | -2.03 | 48.6 |
| Reverse_Archaea_3 | GCA TCT TCT GCC TGT GCT CCC CCG CCA ATT CCT | 33 | 60.6 | -1.72 | 37.1 |
| Reverse_Archaea_4 | GCA TCG CTC AGG AGT GCT CCC CCG CCA ATT CCT | 33 | 63.6 | -2.09 | 48.6 |
| Reverse_Archaea_5 | GCA TCA GGA GTC CGT GCT CCC CCG CCA ATT CCT | 33 | 63.6 | -3.04 | 53.6 |
| Reverse_Archaea_6 | GCA TCC ATG CCT AGT GCT CCC CCG CCA ATT CCT | 33 | 60.6 | -2.07 | 38.9 |
| Reverse_Archaea_7 | GCA TCG TAG AGA GGT GCT CCC CCG CCA ATT CCT | 33 | 60.6 | -1.84 | 38.1 |
| Reverse_Archaea_8 | GCA TCC AGC CTC GGT GCT CCC CCG CCA ATT CCT | 33 | 66.7 | -2.16 | 41.8 |
| Reverse_Archaea_9 | GCA TCT GCC TCT TGT GCT CCC CCG CCA ATT CCT | 33 | 60.6 | -0.94 | 35.8 |
| Reverse_Archaea_10 | GCA TCT CCT CTA CGT GCT CCC CCG CCA ATT CCT | 33 | 60.6 | -0.94 | 35.8 |
| Reverse_Archaea_11 | GCA TCT CAT GAG CGT GCT CCC CCG CCA ATT CCT | 33 | 60.6 | -2.59 | 42.7 |
| Reverse_Archaea_12 | GCA TCC CTG AGA TGT GCT CCC CCG CCA ATT CCT | 33 | 60.6 | -2.86 | 50.9 |

**Supplementary Materials, Table 3. Summary of in-line barcodes theoretical and experimental percentages (mean ± standard deviation).** This table summarizes the theoretical and experimentally observed percentages (mean ± standard deviation) for selected archaeal and bacterial primers. Columns indicate the conditions with zero, one, and two errors, including indels.

| *Domain* | *Orientation* | *Barcode* | *Theoretical %* | *0err_indel* | *0err_noindel* | *1err_indel* | *1err_noindel* | *2err_indel* | *2err_noindel* |
| --- | --- | --- | --- | --- | --- | --- | --- | --- | --- |
| *Bacteria* | Forward | Unknown | 0.00 | 11.67 ± 2.71 | 11.67 ± 2.71 | 0.03 ± 0.01 | 0.30 ± 0.09 | 0.00 ± 0.00 | 0.00 ± 0.00 |
|  |  | Forward_1 | 12.5 | 10.30 ± 1.86 | 10.30 ± 1.86 | 10.74 ± 2.01 | 10.64 ± 2.04 | 11.19 ± 1.54 | 11.75 ± 2.00 |
|  |  | Forward_2 | 12.5 | 11.34 ± 0.93 | 11.34 ± 0.93 | 13.96 ± 0.84 | 14.85 ± 0.81 | 13.07 ± 0.78 | 13.57 ± 1.00 |
|  |  | Forward_3 | 12.5 | 11.51 ± 2.98 | 11.51 ± 2.98 | 13.07 ± 3.16 | 12.59 ± 2.84 | 14.21 ± 3.50 | 15.17 ± 3.07 |
|  |  | Forward_4 | 12.5 | 10.33 ± 1.54 | 10.33 ± 1.54 | 11.76 ± 1.78 | 11.69 ± 1.66 | 10.89 ± 1.54 | 10.92 ± 1.43 |
|  |  | Forward_5 | 12.5 | 11.00 ± 1.65 | 11.00 ± 1.65 | 11.87 ± 1.52 | 11.42 ± 1.43 | 12.87 ± 1.20 | 12.10 ± 1.18 |
|  |  | Forward_6 | 12.5 | 10.27 ± 0.66 | 10.27 ± 0.66 | 13.95 ± 2.48 | 14.66 ± 2.53 | 13.49 ± 2.18 | 12.39 ± 1.53 |
|  |  | Forward_7 | 12.5 | 10.37 ± 0.72 | 10.37 ± 0.72 | 10.86 ± 0.78 | 10.60 ± 0.93 | 10.73 ± 0.80 | 10.70 ± 0.88 |
|  |  | Forward_8 | 12.5 | 13.22 ± 2.13 | 13.22 ± 2.13 | 13.76 ± 2.20 | 13.25 ± 2.08 | 13.55 ± 2.14 | 13.41 ± 2.10 |
|  | Reverse | Unknown | 0.00 | 6.16 ± 2.13 | 6.16 ± 2.13 | 0.01 ± 0.00 | 0.06 ± 0.03 | 0.00 ± 0.00 | 0.00 ± 0.00 |
|  |  | Reverse_1 | 5.88 | 5.63 ± 0.58 | 5.63 ± 0.58 | 7.77 ± 1.72 | 7.68 ± 1.80 | 6.61 ± 1.16 | 7.60 ± 1.51 |
|  |  | Reverse_10 | 5.88 | 6.77 ± 0.56 | 6.77 ± 0.56 | 7.34 ± 0.82 | 7.37 ± 0.69 | 7.11 ± 0.56 | 7.08 ± 0.54 |
|  |  | Reverse_11 | 5.88 | 5.77 ± 0.66 | 5.77 ± 0.66 | 5.55 ± 0.75 | 5.36 ± 0.79 | 6.00 ± 0.61 | 6.01 ± 0.57 |
|  |  | Reverse_12 | 5.88 | 5.22 ± 0.68 | 5.22 ± 0.68 | 5.03 ± 0.67 | 4.86 ± 0.72 | 5.36 ± 0.65 | 5.33 ± 0.60 |
|  |  | Reverse_13 | 5.88 | 5.53 ± 0.80 | 5.53 ± 0.80 | 5.43 ± 0.79 | 5.31 ± 0.76 | 5.63 ± 0.80 | 5.57 ± 0.79 |
|  |  | Reverse_14 | 5.88 | 6.56 ± 2.47 | 6.56 ± 2.47 | 6.46 ± 2.42 | 6.30 ± 2.38 | 7.03 ± 2.63 | 6.87 ± 2.54 |
|  |  | Reverse_15 | 5.88 | 5.92 ± 0.73 | 5.92 ± 0.73 | 5.80 ± 0.74 | 5.67 ± 0.70 | 6.33 ± 0.73 | 5.95 ± 0.72 |
|  |  | Reverse_16 | 5.88 | 5.11 ± 0.67 | 5.11 ± 0.67 | 5.03 ± 0.67 | 4.93 ± 0.65 | 5.23 ± 0.64 | 5.17 ± 0.63 |
|  |  | Reverse_17 | 5.88 | 4.66 ± 0.58 | 4.66 ± 0.58 | 4.64 ± 0.57 | 4.52 ± 0.58 | 5.04 ± 0.46 | 4.73 ± 0.58 |
|  |  | Reverse_2 | 5.88 | 5.21 ± 0.17 | 5.21 ± 0.17 | 5.75 ± 0.86 | 5.59 ± 0.95 | 5.94 ± 0.64 | 5.89 ± 0.61 |
|  |  | Reverse_3 | 5.88 | 5.29 ± 0.30 | 5.29 ± 0.30 | 6.69 ± 0.90 | 6.19 ± 0.72 | 5.88 ± 0.73 | 6.06 ± 0.19 |
|  |  | Reverse_4 | 5.88 | 5.80 ± 0.20 | 5.80 ± 0.20 | 5.68 ± 0.31 | 5.50 ± 0.30 | 6.17 ± 0.31 | 6.06 ± 0.34 |
|  |  | Reverse_5 | 5.88 | 4.92 ± 0.27 | 4.92 ± 0.27 | 5.05 ± 0.32 | 4.76 ± 0.25 | 5.30 ± 0.32 | 5.17 ± 0.32 |
|  |  | Reverse_6 | 5.88 | 6.03 ± 0.28 | 6.03 ± 0.28 | 5.92 ± 0.41 | 5.72 ± 0.33 | 6.35 ± 0.35 | 6.24 ± 0.34 |
|  |  | Reverse_7 | 5.88 | 4.97 ± 0.21 | 4.97 ± 0.21 | 4.85 ± 0.30 | 4.97 ± 0.50 | 5.08 ± 0.19 | 5.04 ± 0.20 |
|  |  | Reverse_8 | 5.88 | 4.95 ± 0.34 | 4.95 ± 0.34 | 7.61 ± 1.70 | 9.97 ± 1.36 | 5.20 ± 0.32 | 5.29 ± 0.34 |
|  |  | Reverse_9 | 5.88 | 5.49 ± 0.23 | 5.49 ± 0.23 | 5.41 ± 0.29 | 5.23 ± 0.35 | 5.74 ± 0.14 | 5.94 ± 0.65 |
| *Archaea* | Forward | Unknown | 0.00 | 9.15 ± 1.97 | 9.15 ± 1.97 | 0.03 ± 0.01 | 2.84 ± 1.10 | 0.00 ± 0.00 | 0.00 ± 0.00 |
|  |  | Forward_1 | 7.69 | 6.87 ± 0.43 | 6.87 ± 0.43 | 7.18 ± 0.45 | 7.11 ± 0.47 | 8.89 ± 0.39 | 8.91 ± 0.36 |
|  |  | Forward_10 | 7.69 | 8.67 ± 0.69 | 8.67 ± 0.69 | 9.29 ± 0.71 | 9.21 ± 0.73 | 8.97 ± 0.70 | 9.39 ± 0.74 |
|  |  | Forward_11 | 7.69 | 6.71 ± 0.58 | 6.71 ± 0.58 | 6.98 ± 0.46 | 6.97 ± 0.40 | 6.99 ± 0.57 | 7.00 ± 0.46 |
|  |  | Forward_12 | 7.69 | 6.42 ± 0.33 | 6.42 ± 0.33 | 7.62 ± 0.27 | 6.63 ± 0.35 | 6.84 ± 0.32 | 6.73 ± 0.34 |
|  |  | Forward_13 | 7.69 | 7.23 ± 0.75 | 7.23 ± 0.75 | 7.46 ± 0.82 | 7.41 ± 0.83 | 7.64 ± 0.84 | 7.53 ± 0.81 |
|  |  | Forward_2 | 7.69 | 7.60 ± 0.36 | 7.60 ± 0.36 | 8.95 ± 1.53 | 7.93 ± 0.50 | 9.20 ± 0.46 | 9.28 ± 1.68 |
|  |  | Forward_3 | 7.69 | 5.95 ± 0.25 | 5.95 ± 0.25 | 6.87 ± 0.26 | 6.37 ± 0.24 | 8.13 ± 1.05 | 6.84 ± 0.28 |
|  |  | Forward_4 | 7.69 | 6.47 ± 0.10 | 6.47 ± 0.10 | 6.92 ± 0.09 | 6.80 ± 0.08 | 7.92 ± 0.31 | 6.82 ± 0.14 |
|  |  | Forward_5 | 7.69 | 6.20 ± 0.47 | 6.20 ± 0.47 | 6.77 ± 0.36 | 6.69 ± 0.30 | 6.91 ± 0.27 | 6.59 ± 0.37 |
|  |  | Forward_6 | 7.69 | 6.10 ± 0.25 | 6.10 ± 0.25 | 6.50 ± 0.21 | 6.69 ± 0.37 | 6.66 ± 0.21 | 6.69 ± 0.30 |
|  |  | Forward_7 | 7.69 | 6.54 ± 0.63 | 6.54 ± 0.63 | 8.13 ± 0.72 | 8.09 ± 0.71 | 6.93 ± 0.61 | 8.24 ± 0.73 |
|  |  | Forward_8 | 7.69 | 9.98 ± 0.83 | 9.98 ± 0.83 | 11.03 ± 0.97 | 11.05 ± 0.93 | 8.58 ± 0.59 | 9.69 ± 0.80 |
|  |  | Forward_9 | 7.69 | 6.13 ± 0.31 | 6.13 ± 0.31 | 6.27 ± 0.30 | 6.20 ± 0.28 | 6.36 ± 0.31 | 6.29 ± 0.30 |
|  | Reverse | Unknown | 0.00 | 11.69 ± 1.34 | 11.69 ± 1.34 | 0.04 ± 0.02 | 0.90 ± 0.48 | 0.00 ± 0.00 | 0.00 ± 0.00 |
|  |  | Reverse_1 | 8.33 | 7.67 ± 0.83 | 7.67 ± 0.83 | 11.41 ± 1.00 | 11.43 ± 1.00 | 8.08 ± 0.87 | 13.06 ± 1.45 |
|  |  | Reverse_10 | 8.33 | 8.22 ± 0.34 | 8.22 ± 0.34 | 8.59 ± 0.33 | 8.61 ± 0.33 | 8.44 ± 0.37 | 8.36 ± 0.37 |
|  |  | Reverse_11 | 8.33 | 7.95 ± 0.16 | 7.95 ± 0.16 | 8.01 ± 0.18 | 8.03 ± 0.19 | 8.16 ± 0.17 | 8.11 ± 0.18 |
|  |  | Reverse_12 | 8.33 | 7.35 ± 0.33 | 7.35 ± 0.33 | 7.43 ± 0.32 | 7.46 ± 0.31 | 7.52 ± 0.32 | 7.54 ± 0.31 |
|  |  | Reverse_2 | 8.33 | 6.54 ± 0.39 | 6.54 ± 0.39 | 8.95 ± 0.71 | 8.90 ± 0.69 | 8.21 ± 0.81 | 7.78 ± 0.46 |
|  |  | Reverse_3 | 8.33 | 8.87 ± 0.52 | 8.87 ± 0.52 | 12.62 ± 1.53 | 11.63 ± 2.09 | 15.71 ± 2.03 | 11.89 ± 2.12 |
|  |  | Reverse_4 | 8.33 | 7.40 ± 0.31 | 7.40 ± 0.31 | 7.52 ± 0.33 | 7.53 ± 0.33 | 7.77 ± 0.33 | 7.56 ± 0.33 |
|  |  | Reverse_5 | 8.33 | 5.71 ± 0.28 | 5.71 ± 0.28 | 6.15 ± 0.28 | 6.20 ± 0.29 | 6.33 ± 0.27 | 6.26 ± 0.31 |
|  |  | Reverse_6 | 8.33 | 7.57 ± 0.30 | 7.57 ± 0.30 | 7.66 ± 0.26 | 7.67 ± 0.24 | 7.78 ± 0.25 | 7.73 ± 0.26 |
|  |  | Reverse_7 | 8.33 | 6.51 ± 0.21 | 6.51 ± 0.21 | 6.69 ± 0.22 | 6.64 ± 0.23 | 6.71 ± 0.24 | 6.65 ± 0.22 |
|  |  | Reverse_8 | 8.33 | 6.67 ± 0.36 | 6.67 ± 0.36 | 6.92 ± 0.33 | 6.96 ± 0.32 | 7.23 ± 0.43 | 6.84 ± 0.35 |
|  |  | Reverse_9 | 8.33 | 7.85 ± 0.20 | 7.85 ± 0.20 | 7.99 ± 0.17 | 8.03 ± 0.19 | 8.04 ± 0.22 | 8.22 ± 0.15 |

**Supplementary** **Materials, Table 4**. **Mapping between numerical identifiers and barcode combinations.**

| Position | Barcode_Combination_Bacteria (Forward-Reverse) | Barcode_Combination_Archaea (Forward-Reverse) |
| --- | --- | --- |
| 1 | 1-1 | 1-1 |
| 2 | 1-2 | 1-2 |
| 3 | 1-3 | 1-3 |
| 4 | 1-4 | 1-4 |
| 5 | 1-5 | 1-5 |
| 6 | 1-6 | 1-6 |
| 7 | 1-7 | 1-7 |
| 8 | 1-8 | 1-8 |
| 9 | 1-9 | 1-9 |
| 10 | 1-10 | 1-10 |
| 11 | 1-11 | 1-11 |
| 12 | 1-12 | 1-12 |
| 13 | 1-13 | 2-1 |
| 14 | 1-14 | 2-2 |
| 15 | 1-15 | 2-3 |
| 16 | 1-16 | 2-4 |
| 17 | 1-17 | 2-5 |
| 18 | 2-1 | 2-6 |
| 19 | 2-2 | 2-7 |
| 20 | 2-3 | 2-8 |
| 21 | 2-4 | 2-9 |
| 22 | 2-5 | 2-10 |
| 23 | 2-6 | 2-11 |
| 24 | 2-7 | 2-12 |
| 25 | 2-8 | 3-1 |
| 26 | 2-9 | 3-2 |
| 27 | 2-10 | 3-3 |
| 28 | 2-11 | 3-4 |
| 29 | 2-12 | 3-5 |
| 30 | 2-13 | 3-6 |
| 31 | 2-14 | 3-7 |
| 32 | 2-15 | 3-8 |
| 33 | 2-16 | 3-9 |
| 34 | 2-17 | 3-10 |
| 35 | 3-1 | 3-11 |
| 36 | 3-2 | 3-12 |
| 37 | 3-3 | 4-1 |
| 38 | 3-4 | 4-2 |
| 39 | 3-5 | 4-3 |
| 40 | 3-6 | 4-4 |
| 41 | 3-7 | 4-5 |
| 42 | 3-8 | 4-6 |
| 43 | 3-9 | 4-7 |
| 44 | 3-10 | 4-8 |
| 45 | 3-11 | 4-9 |
| 46 | 3-12 | 4-10 |
| 47 | 3-13 | 4-11 |
| 48 | 3-14 | 4-12 |
| 49 | 3-15 | 5-1 |
| 50 | 3-16 | 5-2 |
| 51 | 3-17 | 5-3 |
| 52 | 4-1 | 5-4 |
| 53 | 4-2 | 5-5 |
| 54 | 4-3 | 5-6 |
| 55 | 4-4 | 5-7 |
| 56 | 4-5 | 5-8 |
| 57 | 4-6 | 5-9 |
| 58 | 4-7 | 5-10 |
| 59 | 4-8 | 5-11 |
| 60 | 4-9 | 5-12 |
| 61 | 4-10 | 6-1 |
| 62 | 4-11 | 6-2 |
| 63 | 4-12 | 6-3 |
| 64 | 4-13 | 6-4 |
| 65 | 4-14 | 6-5 |
| 66 | 4-15 | 6-6 |
| 67 | 4-16 | 6-7 |
| 68 | 4-17 | 6-8 |
| 69 | 5-1 | 6-9 |
| 70 | 5-2 | 6-10 |
| 71 | 5-3 | 6-11 |
| 72 | 5-4 | 6-12 |
| 73 | 5-5 | 7-1 |
| 74 | 5-6 | 7-2 |
| 75 | 5-7 | 7-3 |
| 76 | 5-8 | 7-4 |
| 77 | 5-9 | 7-5 |
| 78 | 5-10 | 7-6 |
| 79 | 5-11 | 7-7 |
| 80 | 5-12 | 7-8 |
| 81 | 5-13 | 7-9 |
| 82 | 5-14 | 7-10 |
| 83 | 5-15 | 7-11 |
| 84 | 5-16 | 7-12 |
| 85 | 5-17 | 8-1 |
| 86 | 6-1 | 8-2 |
| 87 | 6-2 | 8-3 |
| 88 | 6-3 | 8-4 |
| 89 | 6-4 | 8-5 |
| 90 | 6-5 | 8-6 |
| 91 | 6-6 | 8-7 |
| 92 | 6-7 | 8-8 |
| 93 | 6-8 | 8-9 |
| 94 | 6-9 | 8-10 |
| 95 | 6-10 | 8-11 |
| 96 | 6-11 | 8-12 |
| 97 | 6-12 | 9-1 |
| 98 | 6-13 | 9-2 |
| 99 | 6-14 | 9-3 |
| 100 | 6-15 | 9-4 |
| 101 | 6-16 | 9-5 |
| 102 | 6-17 | 9-6 |
| 103 | 7-1 | 9-7 |
| 104 | 7-2 | 9-8 |
| 105 | 7-3 | 9-9 |
| 106 | 7-4 | 9-10 |
| 107 | 7-5 | 9-11 |
| 108 | 7-6 | 9-12 |
| 109 | 7-7 | 10-1 |
| 110 | 7-8 | 10-2 |
| 111 | 7-9 | 10-3 |
| 112 | 7-10 | 10-4 |
| 113 | 7-11 | 10-5 |
| 114 | 7-12 | 10-6 |
| 115 | 7-13 | 10-7 |
| 116 | 7-14 | 10-8 |
| 117 | 7-15 | 10-9 |
| 118 | 7-16 | 10-10 |
| 119 | 7-17 | 10-11 |
| 120 | 8-1 | 10-12 |
| 121 | 8-2 | 11-1 |
| 122 | 8-3 | 11-2 |
| 123 | 8-4 | 11-3 |
| 124 | 8-5 | 11-4 |
| 125 | 8-6 | 11-5 |
| 126 | 8-7 | 11-6 |
| 127 | 8-8 | 11-7 |
| 128 | 8-9 | 11-8 |
| 129 | 8-10 | 11-9 |
| 130 | 8-11 | 11-10 |
| 131 | 8-12 | 11-11 |
| 132 | 8-13 | 11-12 |
| 133 | 8-14 | 12-1 |
| 134 | 8-15 | 12-2 |
| 135 | 8-16 | 12-3 |
| 136 | 8-17 | 12-4 |
| 137 |  | 12-5 |
| 138 |  | 12-6 |
| 139 |  | 12-7 |
| 140 |  | 12-8 |
| 141 |  | 12-9 |
| 142 |  | 12-10 |
| 143 |  | 12-11 |
| 144 |  | 12-12 |
| 145 |  | 13-1 |
| 146 |  | 13-2 |
| 147 |  | 13-3 |
| 148 |  | 13-4 |
| 149 |  | 13-5 |
| 150 |  | 13-6 |
| 151 |  | 13-7 |
| 152 |  | 13-8 |
| 153 |  | 13-9 |
| 154 |  | 13-10 |
| 155 |  | 13-11 |
| 156 |  | 13-12 |

**Supplementary Materials, Table 6. Summary of selected 96 pairs on in-line barcodes for archaeal and bacterial sets.**

| Domain | Orientation | Set | Names | Sequence (5’ 🡪 3’) |
| --- | --- | --- | --- | --- |
| Archaea | Forward | Set 1 | Forward_Archaea_1 | GCATCCTCTCTAT**CAGCCGCCGCGGTAA** |
|  |  |  | Forward_Archaea_2 | GCATCTATCCTCT**CAGCCGCCGCGGTAA** |
|  |  |  | Forward_Archaea_3 | GCATCGTAAGGAG**CAGCCGCCGCGGTAA** |
|  |  |  | Forward_Archaea_4 | GCATCACTGCATA**CAGCCGCCGCGGTAA** |
|  |  |  | Forward_Archaea_5 | GCATCAAGGAGTA**CAGCCGCCGCGGTAA** |
|  |  |  | Forward_Archaea_6 | GCATCCTAAGCCT**CAGCCGCCGCGGTAA** |
|  |  |  | Forward_Archaea_7 | GCATCCGTCTAAT**CAGCCGCCGCGGTAA** |
|  |  |  | Forward_Archaea_8 | GCATCTCTCTCCG**CAGCCGCCGCGGTAA** |
|  |  |  | Forward_Archaea_9 | GCATCTCGACTAG**CAGCCGCCGCGGTAA** |
|  |  |  | Forward_Archaea_10 | GCATCTTCTAGCT**CAGCCGCCGCGGTAA** |
|  |  |  | Forward_Archaea_11 | GCATCCCTAGAGT**CAGCCGCCGCGGTAA** |
|  |  |  | Forward_Archaea_12 | GCATCGCGTAAGA**CAGCCGCCGCGGTAA** |
|  | Reverse |  | Reverse_Archaea_3 | GCATCTTCTGCCT**GTGCTCCCCCGCCAATTCCT** |
|  |  |  | Reverse_Archaea_4 | GCATCGCTCAGGA**GTGCTCCCCCGCCAATTCCT** |
|  |  |  | Reverse_Archaea_5 | GCATCAGGAGTCC**GTGCTCCCCCGCCAATTCCT** |
|  |  |  | Reverse_Archaea_6 | GCATCCATGCCTA**GTGCTCCCCCGCCAATTCCT** |
|  |  |  | Reverse_Archaea_7 | GCATCGTAGAGAG**GTGCTCCCCCGCCAATTCCT** |
|  |  |  | Reverse_Archaea_8 | GCATCCAGCCTCG**GTGCTCCCCCGCCAATTCCT** |
|  |  |  | Reverse_Archaea_10 | GCATCTCCTCTAC**GTGCTCCCCCGCCAATTCCT** |
|  |  |  | Reverse_Archaea_11 | GCATCTCATGAGC**GTGCTCCCCCGCCAATTCCT** |
| Bacteria | Forward | Set 1 | Forward_Bacteria_1 | GCATCCTCTCTAT**ACTCCTACGGGAGGCAGCAG** |
|  |  |  | Forward_Bacteria_2 | GCATCTATCCTCT**ACTCCTACGGGAGGCAGCAG** |
|  |  |  | Forward_Bacteria_3 | GCATCGTAAGGAG**ACTCCTACGGGAGGCAGCAG** |
|  |  |  | Forward_Bacteria_4 | GCATCACTGCATA**ACTCCTACGGGAGGCAGCAG** |
|  |  |  | Forward_Bacteria_5 | GCATCAAGGAGTA**ACTCCTACGGGAGGCAGCAG** |
|  |  |  | Forward_Bacteria_7 | GCATCCGTCTAAT**ACTCCTACGGGAGGCAGCAG** |
|  |  |  | Forward_Bacteria_8 | GCATCTCTCTCCG**ACTCCTACGGGAGGCAGCAG** |
|  | Reverse |  | Reverse_Bacteria_1 | GCATCTCGCCTTA**GACTACHVGGGTWTCTAAT** |
|  |  |  | Reverse_Bacteria_2 | GCATCCTAGTACG**GACTACHVGGGTWTCTAAT** |
|  |  |  | Reverse_Bacteria_3 | GCATCTTCTGCCT**GACTACHVGGGTWTCTAAT** |
|  |  |  | Reverse_Bacteria_4 | GCATCGCTCAGGA**GACTACHVGGGTWTCTAAT** |
|  |  |  | Reverse_Bacteria_5 | GCATCAGGAGTCC**GACTACHVGGGTWTCTAAT** |
|  |  |  | Reverse_Bacteria_6 | GCATCCATGCCTA**GACTACHVGGGTWTCTAAT** |
|  |  |  | Reverse_Bacteria_7 | GCATCGTAGAGAG**GACTACHVGGGTWTCTAAT** |
|  |  |  | Reverse_Bacteria_8 | GCATCCAGCCTCG**GACTACHVGGGTWTCTAAT** |
|  |  |  | Reverse_Bacteria_9 | GCATCTGCCTCTT**GACTACHVGGGTWTCTAAT** |
|  |  |  | Reverse_Bacteria_10 | GCATCTCCTCTAC**GACTACHVGGGTWTCTAAT** |
|  |  |  | Reverse_Bacteria_11 | GCATCTCATGAGC**GACTACHVGGGTWTCTAAT** |
|  |  |  | Reverse_Bacteria_12 | GCATCCCTGAGAT**GACTACHVGGGTWTCTAAT** |
|  |  |  | Reverse_Bacteria_14 | GCATCGTAGCTCC**GACTACHVGGGTWTCTAAT** |
|  |  |  | Reverse_Bacteria_15 | GCATCTACTACGC**GACTACHVGGGTWTCTAAT** |
|  | Forward | Set 2 | Forward_Bacteria_1 | GCATCCTCTCTAT**ACTCCTACGGGAGGCAGCAG** |
|  |  |  | Forward_Bacteria_2 | GCATCTATCCTCT**ACTCCTACGGGAGGCAGCAG** |

**Supplementary Materials, Table 7. Accession numbers for all of the sequencing data used in this study.**

| *Accession* | *Biosample* | *Title* | *Library ID* | *Files* | *Sample name* |
| --- | --- | --- | --- | --- | --- |
| ERS27189048 | SAMEA120488362 | 16s rRNA sample bacteria 1, protocol with PCR | BACT14_PCR | BACT14_PCR_R1.fastq.gz, BACT14_PCR_R2.fastq.gz | BACT14_PCR |
| ERS27189049 | SAMEA120488363 | shotgun metagenome Zymo standard | Zymo_gDNA | Zymo_gDNA_L1_R1.fastq.gz,  Zymo_gDNA_L1_R2.fastq.gz,  Zymo_gDNA_L2_R1.fastq.gz,  Zymo_gDNA_L2_R2.fastq.gz | metagenomic |
| ERS27189050 | SAMEA120488364 | 16s rRNA sample bacteria 2, protocol with PCR | BACT19_PCR | BACT19_PCR_R1.fastq.gz, BACT19_PCR_R2.fastq.gz | BACT19_PCR |
| ERS27189051 | SAMEA120488365 | 16s rRNA sample bacteria 1, protocol without PCR | BACT14_noPCR | BACT14_noPCR_R1.fastq.gz, BACT14_noPCR_R2.fastq.gz | BACT14_noPCR |
| ERS27189052 | SAMEA120488366 | 16s rRNA sample Zymo equimolar pooling , protocol with PCR | ZYMO_PCR | ZYMO_PCR_R1.fastq.gz, ZYMO_PCR_R2.fastq.gz | ZYMO_PCR |
| ERS27189053 | SAMEA120488367 | 16s rRNA sample Archaea 3, protocol without PCR | ARCH46_noPCR | ARCH46_noPCR_R1.fastq.gz, ARCH46_noPCR_R2.fastq.gz | ARCH46_noPCR |
| ERS27189054 | SAMEA120488368 | 16s rRNA sample Zymo equivolume pooling, protocol with PCR | Zymo_equiV | Zymo_equiV_R1.fastq.gz, Zymo_equiV_R2.fastq.gz | Zymo_equiV |
| ERS27189055 | SAMEA120488369 | 16s rRNA sample Archaea 3, protocol with PCR | ARCH46_PCR | ARCH46_PCR_R1.fastq.gz, ARCH46_PCR_R2.fastq.gz | ARCH46_PCR |
| ERS27189056 | SAMEA120488370 | 16s rRNA sample Zymo equimolar pooling , protocol without PCR | ZYMO_noPCR | ZYMO_noPCR_R1.fastq.gz, ZYMO_noPCR_R2.fastq.gz | ZYMO_noPCR |
| ERS27189057 | SAMEA120488371 | 16s rRNA sample bacteria 3 (Sample 32), protocol without PCR | BACT32_noPCR | BACT32_noPCR_R1.fastq.gz, BACT32_noPCR_R2.fastq.gz | BACT32_noPCR |
| ERS27189058 | SAMEA120488372 | 16s rRNA sample bacteria 3 (Sample 32), protocol with PCR | BACT32_PCR | BACT32_PCR_R1.fastq.gz, BACT32_PCR_R2.fastq.gz | BACT32_PCR |
| ERS27189059 | SAMEA120488373 | 16s rRNA sample Archaea 1, protocol with PCR | ARCH15_PCR | ARCH15_PCR_R1.fastq.gz, ARCH15_PCR_R2.fastq.gz | ARCH15_PCR |
| ERS27189060 | SAMEA120488374 | 16s rRNA sample bacteria 2, protocol without PCR | BACT19_noPCR | BACT19_noPCR_R1.fastq.gz, BACT19_noPCR_R2.fastq.gz | BACT19_noPCR |
| ERS27189061 | SAMEA120488375 | 16s rRNA sample Archaea 2, protocol without PCR | ARCH33_noPCR | ARCH33_noPCR_R1.fastq.gz, ARCH33_noPCR_R2.fastq.gz | ARCH33_noPCR |
| ERS27189062 | SAMEA120488376 | 16s rRNA sample Archaea 2, protocol with PCR | ARCH33_PCR | ARCH33_PCR_R1.fastq.gz, ARCH33_PCR_R2.fastq.gz | ARCH33_PCR |
| ERS27189063 | SAMEA120488377 | 16s rRNA sample Archaea 1, protocol without PCR | ARCH15_noPCR | ARCH15_noPCR_R1.fastq.gz, ARCH15_noPCR_R2.fastq.gz | ARCH15_noPCR |
