# Supplementary material for: A Scalable and Cost‐Effective In‐Line Barcoding Strategy for Standardized 16S rRNA Gene Amplicon Sequencing: Performance Evaluation and Bias Assessment
Source: Mol Ecol Resour. 2026 May 11;26:e70138. doi: 10.1111/1755-0998.70138 (PMC13159520; doi:10.1111/1755-0998.70138)
Supplement: Supplementary file 5 — Data S5: Barcode list for Archaeal and Bacterial primer sets, respectively. Suggested protocol for the use of in‐line barcodes. [file MEN-26-e70138-s001.pdf]

A scalable and cost-effective in-line barcoding strategy for standardized 16S rRNA gene amplicon sequencing: performance evaluation and bias assessment

Detailed ‘How to’ protocole for lib’ prep

1. Accurately measure template DNA concentrations with a spectrophotometer (i.e. Nanodrop) or fluorometer (i.e. QuBit, Quantus, etc). Dilute to 2-5 ng/µL in PCR-grade water to obtain a working concentration. Avoid thawing cycles of template DNA, preferring to store template DNA in aliquots that are only used a limited number of times.

Sample handling is a step that cannot be neglected. How the DNA samples have been extracted, their storage conditions, the A260/A280 ratios and the level of fragmentation of the total DNA must be ensured. Preferably stored at very low temperature, or in a freezer where the temperature remains constant, and suspended in 10mM Tris-HCl pH 7.5 buffer (i.e. Qiagen EB Buffer, cat#19086).

2. Perform the PCR reaction using a polymerase that combines the highest possible fidelity with tolerance to the possible presence of inhibitory substances. Standardize final template DNA concentrations to 0.1 ng per µL of reaction. Use a reaction volume of 35 to 50 µL if possible. Perform preliminary assays to obtain amplicon production on the full range of samples, and play with enzyme and barcoded primers concentrations to reach optimal amplification yields.

The number of PCR cycles naturally depends on the quantity and quality of the template DNA. Do not exceed 30 cycles to avoid significant distortion of the microbial community structure. Ideally, 25 cycles are recommended.

3. The PCR reaction proceeds as follows. A first denaturation step (95°C, 60 sec) is followed by 10 **high stringency cycles** consisting of a denaturation step (95°C, 15 sec), annealing (56°C - Bacteria, 53°C - Archaea, 15 sec), then extension (72°C, 15 sec). These cycles are followed by 20 **low stringency cycles** consisting of a denaturation step (95°C, 15 sec), annealing (60°C, 15 sec), then extension (72°C, 15 sec). The whole process is completed by a final extension (72°C, 45 sec).

This two-phase annealing process favors the annealing of the specific primer section first, then the annealing of the complete primer sequence in the 2nd phase. Be sure to modify the parameters (temperature and time of the extension steps) according to the selected polymerase.
You should also reduce the speed at which your thermocycler works. Typically, most of these machines go from one step to the next at a speed of 6°/sec. To promote primer annealing on environmental DNA, we strongly recommend reducing this rate, at least between the denaturation and annealing steps, to 1°C/sec.

4. Load 3-5 µL of amplicons and amplification controls onto 1.5% agarose gels, and migrate fragments at low voltage (ideally 5 V per cm of gel). On a UV plate reader, take a detailed image, taking care **not to saturate** the signals provided by the amplicons. Manually integrate on a scale of 1 to 3 the signal strength obtained by each amplicon (weak, medium, strong).

Adding a molecular weight scale that is also a mass scale is an advantage if you have the right gel reading device and software. Analysis by capillary electrophoresis (QSep Analyzer, Fragment Analyzer, etc.) would naturally be more accurate… but not necessarily more effective than the good old agarose gel method.

Continued…

5. Pool amplicons in an ‘equimass way’ on the basis of previous integration. For instance, use 1 volume of PCR amplicon for the strong signal, 2 volumes for the medium signal and 3 volumes for the weak signal. This ratio has be adjusted according to the signal strength.

As a general rule, amplicons with widely differing contents should not be pooled. Small variations are acceptable (and normal when using environmental DNA). Large variations, on the other hand, can be induced by the quality of template DNA being too disparate or PCR issues. This can lead to significant distortions in community structure.

6. Perform a single purification of the pooled amplicons with magnetic beads according to the information supplied by the manufacturer of your preferred material.

7. Accurately measure the concentration of the purified amplicons with a spectrophotometer or fluorometer and calculate the final library concentration in nMole or pMole.

Typically, this concentration is required at the nMole or pMole scale prior to sequencing, which can be calculated using online tools (e.g. using the NEBBioCalculator, https://nebiocalculator.neb.com).
